# Structures of *Mycobacterium smegmatis* 70S ribosomes in complex with HPF, tmRNA, and P-tRNA

**DOI:** 10.1038/s41598-018-31850-3

**Published:** 2018-09-11

**Authors:** Satabdi Mishra, Tofayel Ahmed, Anu Tyagi, Jian Shi, Shashi Bhushan

**Affiliations:** 1 School of Biological Sciences, Nanyang Technological University, Singapore, Singapore; 2 Center for BioImaging Sciences, National University of Singapore, Singapore, Singapore; 3 NTU Institute of Structural Biology, Nanyang Technological University, Singapore, Singapore

## Abstract

Ribosomes are the dynamic protein synthesis machineries of the cell. They may exist in different functional states in the cell. Therefore, it is essential to have structural information on these different functional states of ribosomes to understand their mechanism of action. Here, we present single particle cryo-EM reconstructions of the *Mycobacterium smegmatis* 70S ribosomes in the hibernating state (with HPF), trans-translating state (with tmRNA), and the P/P state (with P-tRNA) resolved to 4.1, 12.5, and 3.4 Å, respectively. A comparison of the P/P state with the hibernating state provides possible functional insights about the Mycobacteria-specific helix H54a rRNA segment. Interestingly, densities for all the four OB domains of bS1 protein is visible in the hibernating 70S ribosome displaying the molecular details of bS1-70S interactions. Our structural data shows a Mycobacteria-specific H54a-bS1 interaction which seems to prevent subunit dissociation and degradation during hibernation without the formation of 100S dimer. This indicates a new role of bS1 protein in 70S protection during hibernation in Mycobacteria in addition to its conserved function during translation initiation.

## Introduction

Ribosomes are the protein synthesis machineries of the cell, which translate genetic codes on mRNA into protein in all living species. Cryo-electron microscopy (cryo-EM) in the recent decade has aided in many functional studies on the ribosome by providing various snapshots of ribosome translation^1–4^. While the ribosome core is structurally conserved, species-specific domains are widely observed across ribosome structures among prokaryotes and eukaryotes.

Stationary phase of bacteria has declined growth due to the unfavourable conditions such as the lack of nutrients^5^. In stationary phase, ribosome can exist in the inactive hibernating state instead of dissociating into individual subunits. Hibernating ribosomes may exist either as a monomeric 70S or a dimer of 70S known as 100S^5–10^. The hibernation state of ribosome is driven by Stationary phase induced Ribosome Associated proteins (SRA proteins) in bacteria, which include hibernation promoting factor (HPF), ribosome modulation factor (RMF), YfiA, and plastid pY factor^6,11^. HPF exists in two variants in bacteria, as HPF^short^ with only one domain and HPF^long^ with an additional CTD to HPF^short^ ^5,7,10^. HPF^short^ (and N-terminal domain (NTD) of HPF^long^) is capable of inactivating 70S ribosome by binding to the small subunit (SSU) at the inter-subunit space^5^. However, it needs another SRA protein RMF in *E. coli (Ec)* for the formation of 100S. Recent structures of 100S ribosomes from *Staphylococcus aureus* (*Sa*) and *Lactococcus lactis* (*Ll*) revealed that HPF^long^ is responsible for ribosome dimerization without RMF^7,10^.

Bacteria (unlike eukaryotes) lack the mRNA proofreading before translation, resulting in the translation of defected or truncated mRNAs which might stall the translating ribosomes. To rescue these stalled ribosomes in bacteria a specialized RNA species, known as transfer-messenger RNA (tmRNA), binds to ribosome, rescues them from stalling and resumes translation^12–15^. Studies have corroborated the fact that trans-translation state of the ribosome in bacteria helps to overcome the effects of ribosome-targeting anti-microbial agents^13,16^. Unlike in *Ec*, the tmRNA encoding gene, *ssrA* is essential in *M. tuberculosis* (*Mtb*)^17^.

Here, we have used single particle cryo-EM reconstruction to determine the endogenous structures of *M. smegmatis (Ms)* 70S ribosome in the hibernating state, trans-translating state, and P-tRNA bound P/P state. During the course of this work three structures of 70S ribosomes in P/P state from Mycobacteria, two from *Ms*^18,19^ and one from *Mtb*^20^, were published. While, our 70S model of P/P state is consistent with their models, the hibernating and the trans-translating states provide new insights about mycobacterial ribosome. We observe a novel interaction of *Ms* bS1 protein with Mycobacteria-specific helix H54a in the hibernating state indicating an additional function of bS1 in stabilization and protection of the *Ms* 70S ribosome during stationary/dormant phase.

## Results and Discussion

**Figure 1:**
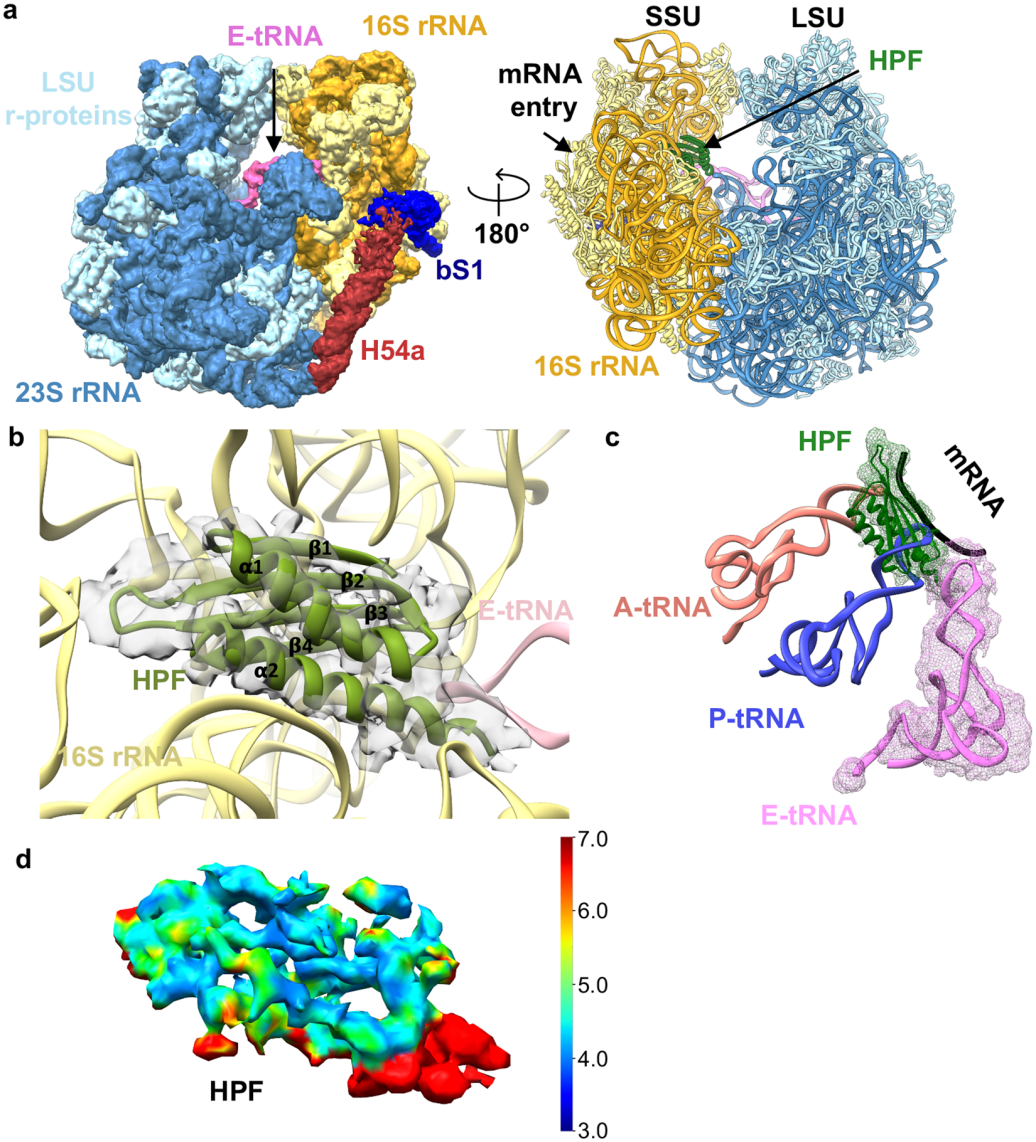
Structure of the hibernating state of *Ms* 70S ribosome. (**a**) Cryo-EM map of the hibernating state of 70S ribosome at 4.1 Å resolution (left) along with the model (right). LSU r-proteins are colored in light-blue, LSU rRNAs in steel-blue, SSU r-proteins in light-yellow, SSU rRNAs in golden-yellow, H54a in deep red, bS1 in deep-blue, E-tRNA in pink, and HPF in green. The densities for H54a and bS1 are shown at threshold 0.025 and for the rest at threshold 0.04 in UCSF chimera^55^. (**b**) A model of the NTD of *Ms* HPF with density in green mesh. HPF binds to the mRNA channel at the inter-subunit space, surrounded by 16S rRNA and E-tRNA, adopting the conserved β_1_-α_1_-β_2_-β_3_-β_4_-α_2_ arrangement. (**c**) HPF occupies the A- and P-tRNA binding sites in SSU. Models of mRNA (black), A-tRNA (salmon), and P-tRNA (blue) from *Ec* (PDB: 5AFI)^60^ are super-positioned with HPF to show the steric clashes. (**d**) Resmap calculation for HPF density in the hibernating state 70S map showing the local resolution in Å as indicated in the scale bar.

Ribosomes were prepared from *Ms* (mc^2^ 155) cells harvested at the log (70S-log) and stationary phases (70S-stat) to obtain the 70S ribosomes in different functional states. The 70S-log cryo-EM dataset resulted in one major class of 70S ribosome (with P-tRNA, 99.4% particles) resolved to 3.4 Å (the P/P state) and one minor class of trans-translating 70S ribosome with the presence of a tmRNA-SmpB (0.6% particles) resolved to 12.5 Å (Supplementary Figs S1–S3). The 70S-stat cryo-EM dataset resulted in one major class of 70S ribosome resolved to 4.1 Å 70S map (Supplementary Fig. S3). When compared with the P/P state 70S map, additional density for HPF is clearly present in this map at the inter-subunit space thus representing the hibernating state of 70S (Fig. 1).

*Ms* ribosome shares the conserved ribosomal architecture with *Ec* ribosome. The small subunit (SSU) has a total of 21 r-proteins and one rRNA molecule, 16S rRNA. However, in contrast to *Ec* SSU, protein bS21 is missing in *Ms* and one additional protein identified as bS22 is present near the decoding center (DC) keeping the overall number of r-proteins in *Ms* SSU similar to *Ec* (Supplementary Table S1). The large subunit (LSU) has 35 r-proteins and two rRNA molecules, 23S rRNA, and 5S rRNA. Compared to *Ec*, *Ms* LSU has one additional protein bL37 located near the peptidyl transferase center (PTC). There are three major insertions in domain I, II, and III which have altered the topology of the 23S rRNA structure in mycobacterial ribosome. The 113 nucleotides long insertion near the mRNA exit site, helix H54a , is the most prominent feature of the mycobacterial ribosome.

### Structure of *M. smegmatis* ribosome in the hibernating state

HPF^long^ mediated formation of 100S dimers has been reported in *T. thermophilus* (*Tth*), *Sa, Ll*, and *B. subtilis* (*Bs*), but not in Mycobacteria although they possess HPF^long^ variant with significant sequence identity with other HPF^long^ ^5-7,10,11^ (Supplementary Fig. S4). HPF was first characterized as protein Y (pY, Rv3241c) in *Mtb*^11^. Our HPF-70S ribosome exists in an un-rotated state of ribosome similar to *Sa* HPF-70S (PDB ID: 5ND8) and *Tth* HPF-70S (PDB ID: 4V8G)^6,8^. The NTD of *Ms* HPF adopts the conserved β_1_-α_1_-β_2_-β_3_-β_4_-α_2_ arrangement and is located between the head and body of the SSU in a pocket formed by 16S rRNA helices (Fig. 1b) at the inter-subunit space. As the density of HPF is moderately resolved in the hibernating state 70S map, sequence analysis was carried out to understand the mode of interaction between HPF and SSU. The residues involved in the stacking interaction between β-sheets and 16S rRNA helices are conserved in the NTD of *Ms* HPF between Lys36/Arg38 and U947, and Glu100 and G948 at the head of SSU. Super-position of A- and P-tRNA models into our map depicts how HPF physically occupies and blocks the A- and P-tRNA binding sites at the mRNA channel (Fig. 1c) making these ribosomes translationally inactive. Conserved residues (Lys 53, Arg 56, and Arg 59) of helix α_2_ overlap with the position of phosphate backbones of A- and P-tRNA at the body of SSU. This shows how HPF could block binding of certain antibiotics like hygromycin B, tetracycline, and edeine targeting A- and P-tRNA binding sites on the 70S^21^. No density for the first 30 amino acids as well as for the CTD of HPF is visible in our map suggesting either they are flexible in nature or they might be involved some additional but yet unknown translational functions. A BLASTp search across the bacteria failed to identify any homologs for the first 30 residues of *Ms* HPF^22^. Interestingly, 100S dimer formation has not been reported in Mycobacteria and our biochemical analysis of the total ribosomes purified from the log (24 h), stationary (48 h) and extended stationary phase (66 h) under both the low (50 mM) and high salt (500 mM) conditions showed intact 70S ribosomes without any detectable presence of 100S dimers in *Ms* (Supplementary Fig. S5). This further supports that the *Ms* ribosomes exist as stable 70S particles without forming 100S dimer during hibernation. A sequence comparison of the CTD of HPF^long^ from *Ms, Mtb, Sa*, and *Ll* shows that one out of the five conserved residues proposed to be involved in the formation of 100S dimers is not conserved in Mycobacteria (Supplementary Fig. S6h). Whether this is the reason that 100S dimer is not formed in *Ms* would need further investigations. This also raises the possibility that C-terminal domain (CTD) of HPF might be involved in some other unknown functions.

**Figure 2:**
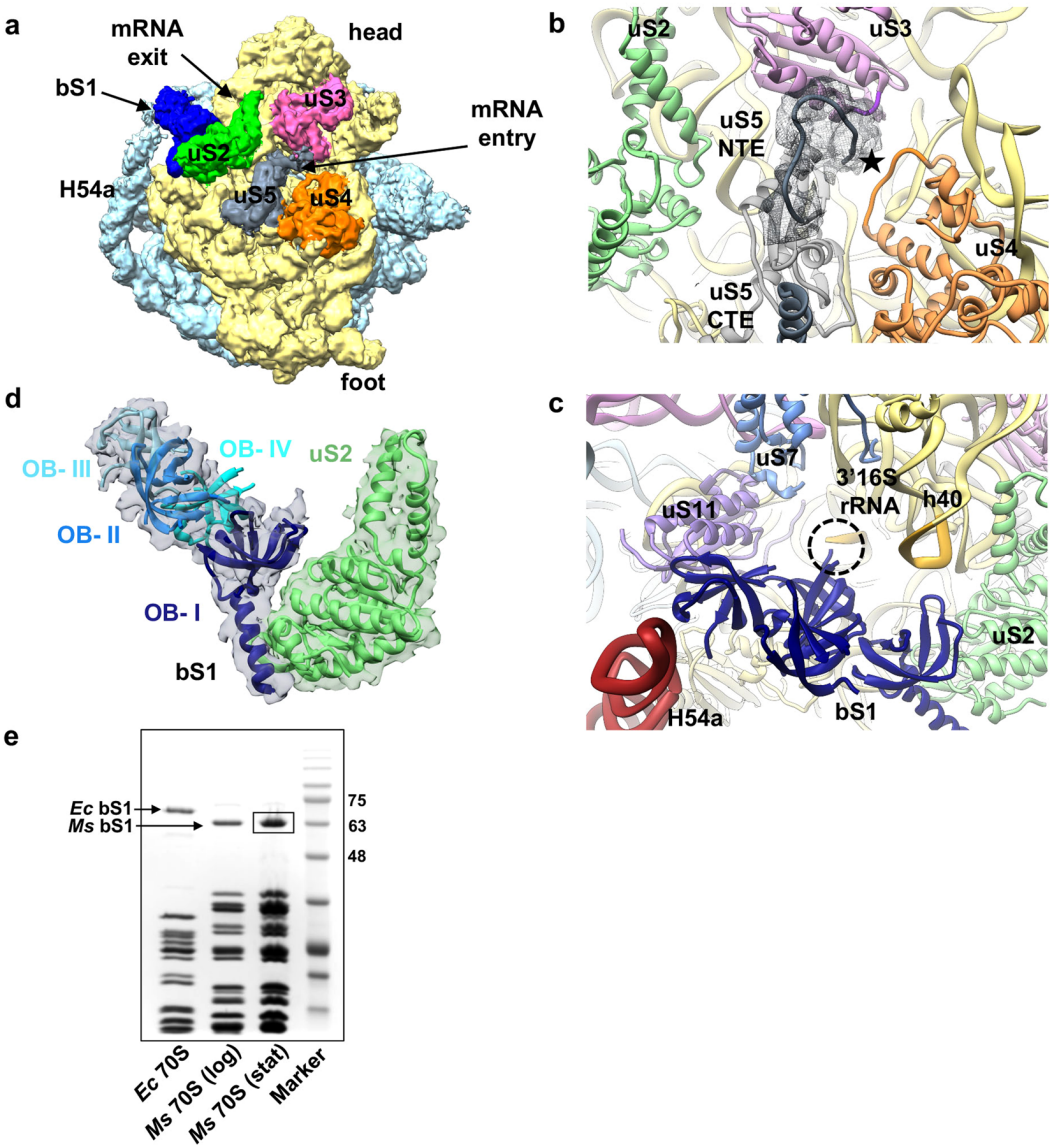
Architecture of mRNA entry and exit sites in the hibernating state of *Ms* 70S ribosome. (**a**) A surface view of the SSU in hibernating state of 70S shown from the solvent-side. Structural landmarks of SSU and the proteins around the mRNA entry and exit sites are labelled as indicated. The density for bS1 is shown at threshold 0.025 and for the rest at threshold 0.04 in UCSF chimera^55^. (**b**) mRNA entry site in *Ms* is formed by three r-proteins, uS3, uS4, and uS5. The uS5 NTE extends towards the mRNA entry channel (marked with a star symbol) and CTE forms an α-helix on the SSU surface. Density of uS5 NTE is shown in grey mesh. (**c**) mRNA exit site (marked by a dotted circle) in *Ms* is formed by two r-proteins uS7 and uS11, and 16S rRNA. bS1 protein is present near to the mRNA exit. The N-terminal α-helix of *Ms* bS1 interacts with uS2 and one of the OB domain (domain III) with H54a. bS1 is colored in deep blue, uS2 in green, uS3 in pink, uS4 in orange, and uS5 in grey colors in (**a**–**c**). (**d**) Zoomed-in view of the bS1. Density for bS1 is in transparent grey, the four OB domains of bS1 are labelled as indicated and highlighted in different shades of blue. Densities for OB domains and uS2 protein are shown at the different thresholds for clarity. (**e**) 15 pmol of 70S ribosomes from *Ec*, *Ms* (log phase), and *Ms* (stationary phase) were analysed on 12% SDS-PAGE with coomassie blue staining. The bS1 protein is highlighted. *Ms* 70S (stationary phase) bS1 protein band (boxed) is further analysed by mass spectrometry (Supplementary Fig. S9).

Interestingly, a strong density is present near the mRNA exit site adjacent to the uS2 protein which further extends towards H54a and anti-SD (anti-Shine Dalgarno) sequence of 16S rRNA at lower threshold. NTD of bS1 fitted well in this density whereas there is still unaccounted density after fitting CTD of HPF in this density (Supplementary Figs S7 and S8). Therefore, this density was assigned to bS1 protein. This is also consistent to the earlier localization of bS1 at this position across various ribosome structures^23–26^ (Supplementary Fig. S8). The presence of bS1 in the stationary phase ribosomes was also confirmed by the SDS-PAGE analysis of the 70S-stat ribosomes (Fig. 2e) followed by the identification with mass spectrometry analysis (Supplementary Fig. S9).

bS1 is the largest and most acidic protein of *Ms* SSU comprising of 449 residues. *Ms* bS1 (similar to bacterial S1 protein) belongs to the oligonucleotide/oligosaccharide binding (OB)-fold superfamily of proteins and has four OB domains in contrast to six OB domains in *Ec*^27^. The density of bS1 in our map could accommodate all the four predicted OB domains for *Ms* bS1. uS2 interacting N-terminal α-helix and neighbouring first OB domain of bS1 (NTD) could be fitted unambiguously into this density (Supplementary Fig. S7b) while three remaining OB domains were manually fitted into the remaining fragmented density (Fig. 2d). This is further supported by the earlier reports of the N-terminal α-helix of bS1 interacting with uS2 by salt bridges^25,28^. In addition to this, the first OB domain also shares surface contact with 16S rRNA (h40) in our HPF-70S map. These interactions therefore, contribute towards the well-resolved density of bS1 NTD in our HPF-70S EM map and agrees with the assumption that NTD of bS1 is the anchoring domain to the ribosome^29^. bS1 has not been seen earlier in any of the hibernating state of ribosomes from *Ec*, *Tth*, *Sa*, and *Ll* while there is clear well-ordered density of bS1 in our hibernating map of *Ms*. Super-positioning of the 70S models of *Sa* and *Ll* 100 dimers (PDB ID: 5NG8 and 5MYJ) with our HPF-70S map revealed that the first OB domain of bS1 in our HPF-70S map would sterically overlap with the CTD of *Sa* and *Ll* HPF^long^ in those ribosome dimers^7,10^. The CTDs of both HPFs in *Sa* and *Ll* (interacting with the uS2) are localized at the same position as the first OB domain of bS1 in our HPF-70S map (Supplementary Fig. S6). Thus, it is possible that because of the presence of the *Ms* bS1 protein at this location, the *Ms* ribosome does not dimerize in a 100S state similar to *Sa* and *Ll* although they possess HPF^long^. This also suggests a direct role of *Ms* bS1 in ribosome resuscitation from the inactive/hibernating state which might be advantageous for slow-growing Mycobacteria. Ribosomes in a 100S state is more stable than the 70S as it prevents subunit dissociation and rRNA degradation. Since 100S state has not been observed in Mycobacteria^11^ and our biochemical analysis also did not detect any 100S formation (Supplementary Fig. S5), it might be disadvantageous to these organisms unless some other mechanism is employed for stabilization of 70S in hibernating state in Mycobacteria. Therefore, it seems plausible that *Ms* bS1 could provide stability to the HPF bound hibernating 70S ribosome in Mycobacteria without forming 100S in addition to its normal function during translation initiation. It is also possible that the presence of the *Ms* bS1 near the mRNA exit site could help in rapid translation initiation by efficiently positioning the transcripts for translation during the recovery from the hibernation. This could be particularly important for the translation of leaderless mRNAs present in Mycobacteria as *Ms* bS1 is significantly different compared to *Ll* and *Sa* (Supplementary Fig. S10). Thus, both bS1 and H54a at this location could help in ribosome resuscitation from the inactive state of ribosome. The presence of strong density of bS1 in our HPF-70S map can be further explained by its specific interaction with H54a during hibernation as discussed in further sections.

### Structure of *M. smegmatis* tmRNA bound 70S ribosome in the trans-translating state

**Figure 3:**
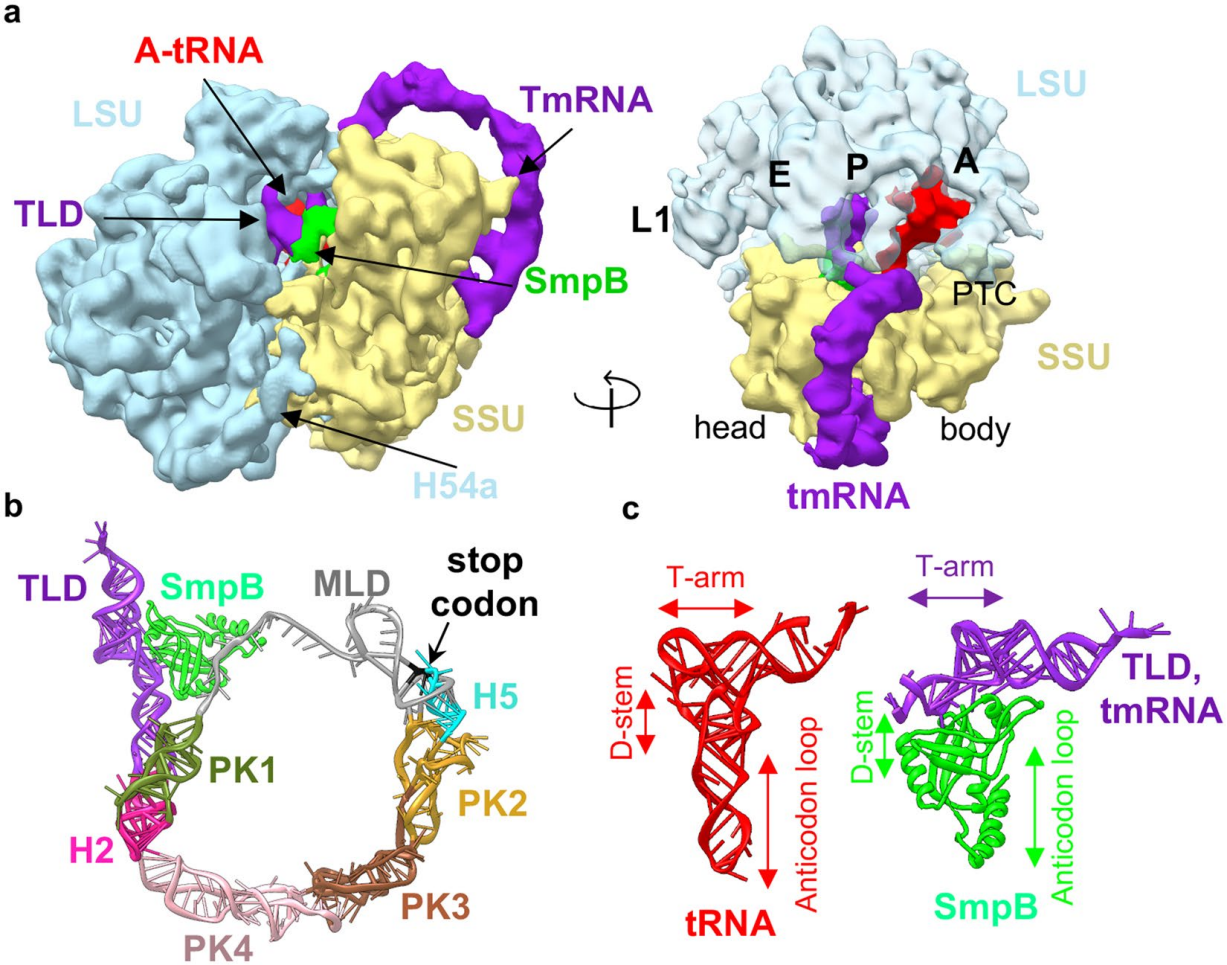
Structure of the trans-translating state of *Ms* 70S ribosome. (**a**) Cryo-EM map of the tmRNA-SmpB complex bound 70S ribosome at 12.5 Å resolution. Density is shown as surface. tmRNA is shown in purple, SmpB in green, and A-tRNA in red (left). LSU in transparent blue showing the tmRNA-TLD and SmpB occupying the P-site and tRNA occupying A-site (right). (**b**) Architecture of tmRNA and SmpB when bound to the 70S. TLD is shown in purple, psuedoknots (PK) 1–4 are in deep green, pink, brown, and yellow, respectively. Helix 2 (H2) is in magenta, helix 5 (H5) in light blue, MLD in grey (stop codon in black), and SmpB in green. (**c**) Comparison of the structure of a typical tRNA with tmRNA-TLD and SmpB complex showing how tmRNA (TLD) and SmpB complex mimics a tRNA.

*Ms* tmRNA is 369 nt long and shares 50.7% sequence identity with *Tth* (349 nt) and 51.5% with *Ec* (363 nt). Secondary structure of *Ms* tmRNA is presented in the Supplementary Fig. S11. tmRNA is consisted of four pseudoknots (PK 1–4), two helices (H2 and H5), one tRNA like domain (TLD), and one mRNA like domain (MLD) (Fig. 3b)^12,14^. The four pseudoknots of tmRNA form an arc-like structure reaching from the beak of SSU to the mRNA entry channel while the two helices (H2 and H5) help in the accurate positioning of tmRNA on to the ribosome. The TLD is formed by the 5′ and 3′ ends of tmRNA and together with the small protein B (SmpB) mimic the tRNA structure (Fig. 3c). SmpB is a conserved protein in prokaryotes and an essential component of trans-translation^12,14^. This 18.2 kDa basic protein (pI: 10.27) stabilizes tmRNA binding at the A- and P-tRNA binding sites and compensates for the D-stem and the anticodon loop of tRNA in tmRNA and is an indispensable element in trans-translation (Fig. 3c). The tmRNA MLD is the most crucial part of tmRNA (gray color in Fig. 3b). This small stretch of RNA mimics mRNA during trans-translation in ribosome. The degradation tag encoded by MLD is 12 residues long (ADSNQRDYALAA) in *Ms* (*Ec* - ANDENYALAA, *Tth* - ANTNYALAA) and therefore carries a short insertion in MLD to accommodate 2–3 additional codons to avoid any steric or topologic constrains when compared with *Ec and Tth* (Supplementary Fig. S12). Length of the degradation tag varies from 8–35 residues with a nearly conserved 3′ end in most bacteria except in mycoplasma^30^. The binding, translation and translocation of the tmRNA-SmpB complex in trans-translation is similar to the amino-acylated tRNA in translation^12,14,15^.

Surprisingly, we were able to identify this minor population of about 0.6% of 70S ribosome in trans-translational state in the 70S-log dataset. We believe that use of a bigger dataset of about 391,000 particles with a careful 3D classification allowed us to identify this state. Because of the limited number of particles (~1,300), the trans-translating map could only be refined to an average resolution of 12.5 Å (Supplementary Figs S1 and S3). Docking of the known tmRNA bound 70S models of *Ec* and *Tth* in our map enabled us to identify that our trans-translation state represents the resumed state with tRNA bound at A-site and tmRNA-SmpB complex at P-site (Fig. 3a,b)^12,15,31,32^. It is possible that due to the abundance of leaderless mRNA (about one fourth of total mRNA)^33^, which might result in higher level of translational stalling because of the incorrect translation initiation of these classes of transcripts, trans-translation state plays significant role during translation in Mycobacteria which would need further investigations.

### Structure of the *M. smegmatis* 70S ribosome in the P/P state

**Figure 4:**
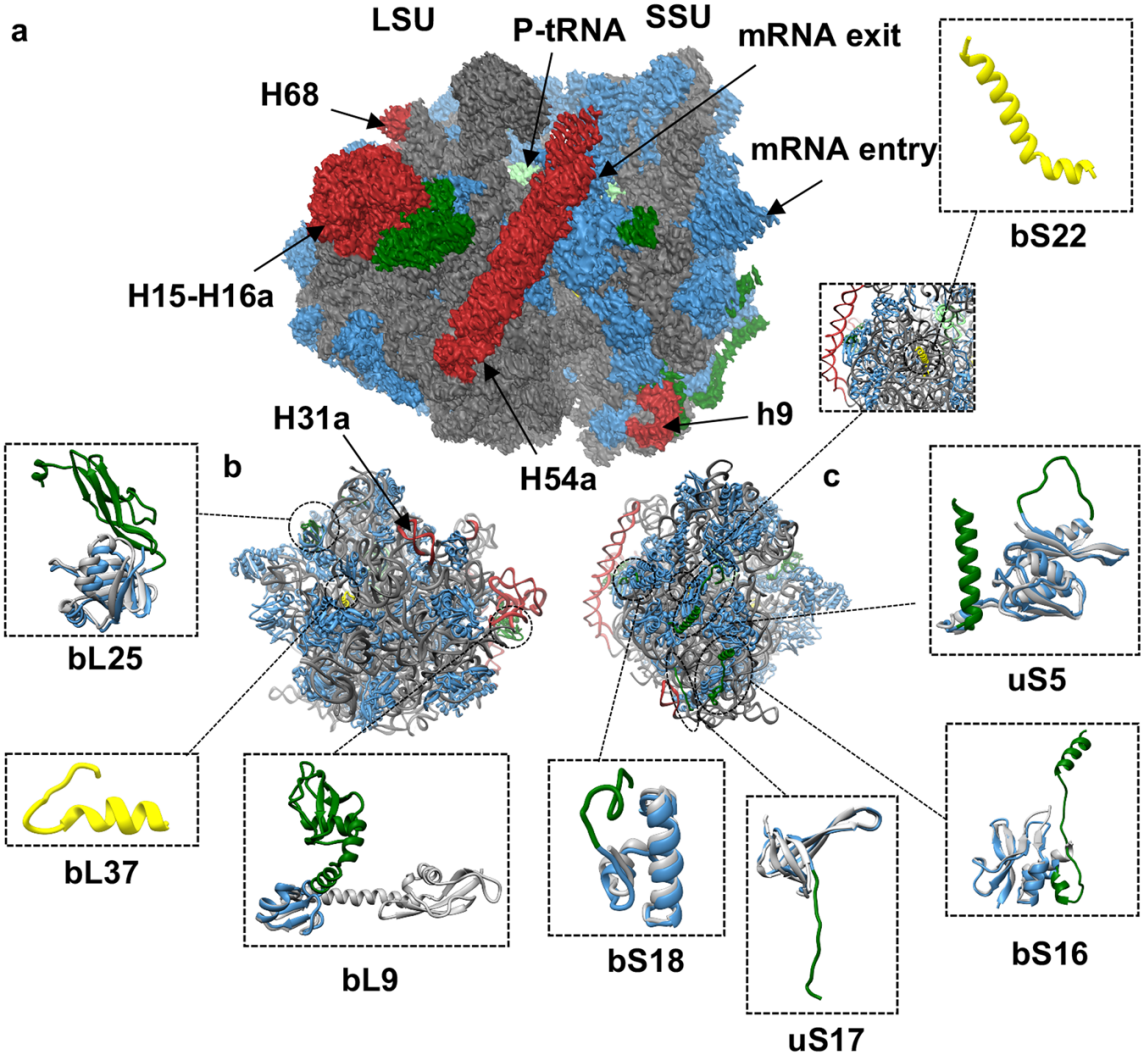
Unique structural features of the *Mycobacterium smegmatis* (*Ms*) 70S ribosome. (**a**) Cryo-EM map of *Ms* 70S ribosome at 3.4 Å resolution in the non-rotated P/P state. Density is shown as surface and colored as - rRNA: grey, r-proteins: blue, and P-tRNA: light green. *Ms*-specific rRNA and r-protein extensions (in comparison to *Ec*, PDB: 4YBB)^64^ are shown in deep red and deep-green, respectively. (**b**) LSU model of *Ms* 70S from the solvent-side. *Ms*-specific protein extension of bL25 and repositioning of bL9 (in green) are shown in the zoomed view (blue color) with superimposition of respective *Ec*-homologs in grey. Mycobacteria-specific protein bL37 is colored in yellow. (**c**) SSU model of *Ms* 70S from the solvent-side. *Ms*-specific protein extensions for uS5, bS16, uS17, and bS18 (in green) are shown in zoomed view (blue color) with superimposition of respective *Ec*-homologs in grey. Mycobacteria-specific protein bS22 is colored in yellow.

With a conserved core structure of ribosome, mycobacterial ribosome exhibits various unique extensions in its rRNAs and r-proteins (Fig. 4 and Supplementary Table S1). The C-terminal extension (CTE) of uS5 forms an extra α-helix which is localized on the SSU surface (Figs 2b and 4). The CTE of bS16 (30 residues out of 74 residues) is extended to form a short helix away from the core of bS16 where it reaches and interacts with uS4. The N-terminal extension of *Ms* uS17 (modeled residues: Gly 6 to Gly 18) extends toward helix h9 of 16S rRNA to probably stabilize the 8 nt insertion (185G–202G) of h9 in Mycobacteria. A Mycobacteria-specific protein bS22 is observed near the DC, as also reported by Hentchel J, *et al*.^19^ (Fig. 4). It occupies a similar position as of the eukaryotic ribosomal protein eL41 in 80S ribosome from *S. cerevisiae* and mitochondria ribosomal protein mL41 in *S. cerevisiae*^34,35^.

In *Ms* LSU, bL25 is more than double in size (215 residues) compared to its *Ec* homolog (94 residues) because of its additional CTD (Fig. 4). The NTD interacts with the loop E of 5S rRNA and binds to the ribosome to form a part of the central protuberance^36^. The structure of *Ms* bL25 CTD is similar to *Tth* bL25 CTD and might assist in tRNA proofreading in Mycobacteria^37^. One additional protein bL37 was localized near the PTC as observed recently^18,19^. However, the exact function of bL37 remains to be elucidated.

The rRNA extensions of the *Ms* LSU are the most striking features of the mycobacterial ribosome. *Ms* H15 (A272–U318) is extended by 36 nt in comparison to *Tth* H15 (absent from *Ec*). H15 and H16a are the two complementary RNA helices exhibiting RNA kissing loop interaction in mycobacterial ribosome as also observed in the recent mycobacterial 70S ribosome structures^18–20^. RNA kissing loop interactions provide unusual RNA stability in RNA molecules (as seen in human immunodeficiency virus, HIV) and such stable interactions will therefore, be more interesting to investigate in pathogenic Mycobacteria^38^. Interestingly, topology of bL9 protein in this region is altered possibly to accommodate changes in H15 and H16a. A 23 nt long insertion at position U748 in *Ms* H31 results in a new helix H31a. This insertion is present at the base of the central protuberance and extends to interact with bL27.

**Figure 5:**
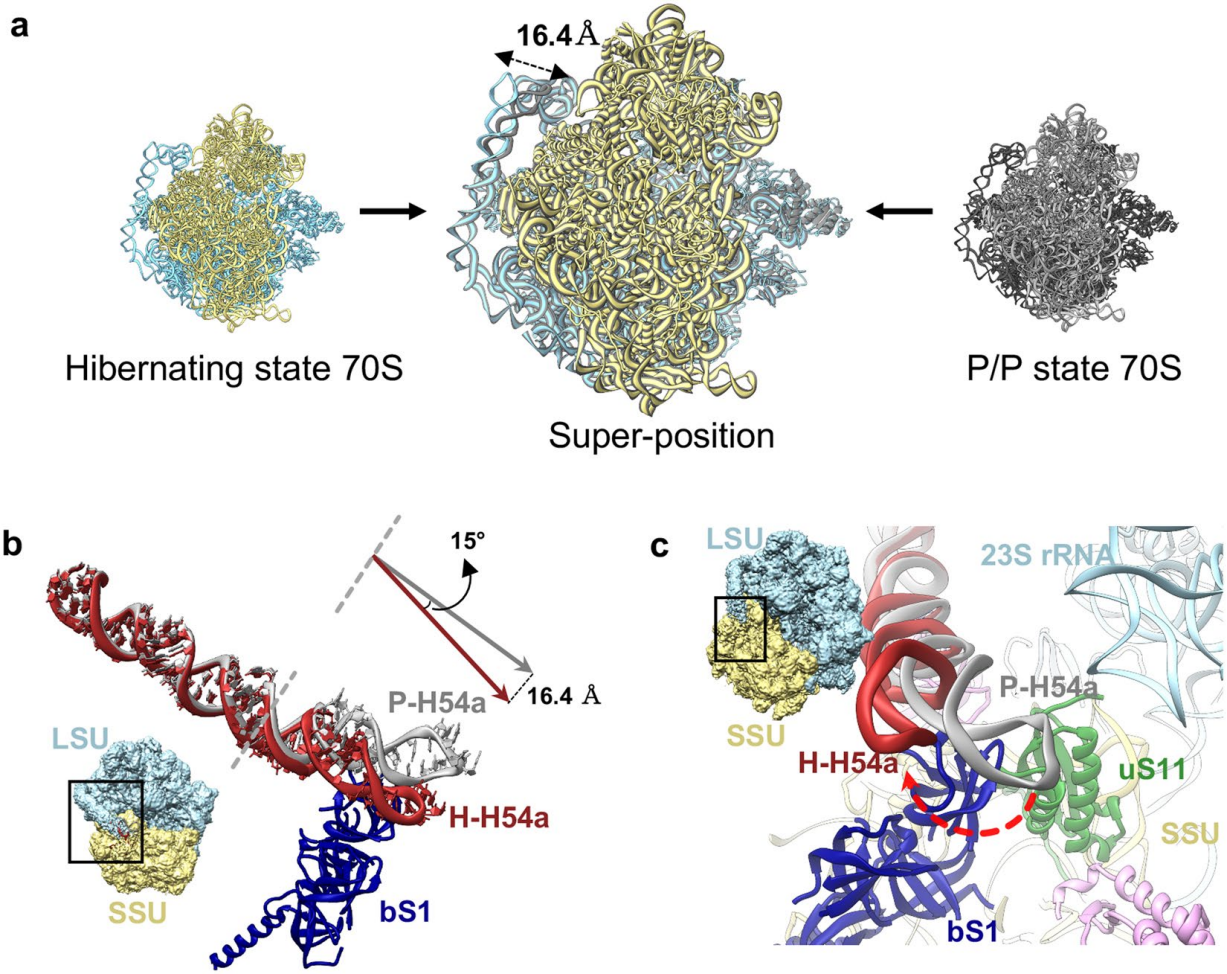
Movement of H54a rRNA segment between the P/P state and hibernating state of the 70S ribosome. (**a**) Super-position (centre) of hibernating ribosomal model in left (LSU in blue and SSU in yellow) with P/P state ribosomal model in right (LSU in dark grey and SSU in light grey) shows the H54a movement. (**b**) H54a movement between the two states. The H54a tip moves about 16.4 Å with 15° angular tilt towards the bS1 protein in the hibernating state. P-H54a in grey: H54a in P/P state and H-H54a in deep red: H54a in hibernating states. (**c**) H54a movement in the hibernating state towards bS1. The density maps in the snippet (in b and c) represent the orientation of the 70S.

H54a is a Mycobacteria-specific 113 nt long insertion in H54 at position G1532 which emerges at the solvent side of LSU near the L1 stalk and unequivocally is the most prominent feature of mycobacterial ribosomes (Fig. 4a). H54a is highly flexible and the observed density is fragmented near the tip of the helix in the P/P state 70S ribosome (Supplementary Fig. S2c). Its close proximity to the mRNA exit site and proteins bL9, bS6, and uS11 indicates its possible function in translation initiation and regulation. *Ms* bL9 is flipped towards H54a and bS6 shares surface contact with H54a. Interestingly, H54a can be seen directly interacting with the bS1 in close vicinity of the mRNA exit site in the hibernating state (Fig. 5).

### Structure of mRNA entry and exit sites in *M. smegmatis* 70S ribosome

Ribosome function is defined by the translation of mRNAs into nascent polypeptide chains. Interestingly, one-fourth of the total mRNAs, so-called the leaderless mRNAs, in Mycobacteria are devoid of SD sequences^33^. Therefore, Mycobacteria-specific remodeling of the mRNA binding and recognition sites might be required for the efficient translation initiation. mRNA binding and recognition during translation initiation is generally facilitated by the mRNA entry and exit sites together with bS1 on the SSU^25,28,39^. mRNA entry site in *Ms* is composed of three proteins, uS3, uS4, and uS5. The uS5 NTE extends into the mRNA entry channel where it might interact with mRNAs of leaderless transcripts to support correct positioning of the first codon since uS5 functions in translational fidelity^40^.

mRNA exit site holds eminent importance in translation initiation as it harbours the interaction between SD and anti-SD sequences. *Ec* mRNA exit site is composed of uS7, uS11, bS18, and bS21. *Ms* mRNA exit site superimposes well with the *Ec* with the exception of 10 residues long NTE in bS18 and the absence of bS21 (not present in *Ms* genome) (Fig. 2 and Supplementary Fig. S13). bS21 is a non-essential and late ribosome binding protein^41,42^. The stable localization of *Ms* bS1 near the mRNA exit site validates its function in the mRNA binding and recognition. bS1 interacts with Mycobacteria-specific H54a of 23S rRNA, and this interaction seems to stabilize both bS1 and H54a in the close vicinity of the mRNA exit site (Fig. 2c). Interestingly, a partial density of bS1 is also observed in our P/P state 70S map when filtered to 10 Å (Supplementary Fig. S14). The N-terminal α-helix of bS1 could be fitted into this density and first OB domain only appears at low threshold while rest of the bS1 is not visible. This suggests that bS1 is present during both the P/P and the hibernating states in *Ms* as opposed to the general proposal of an on- and -off mechanism of bS1 during translation initiation and elongation^43^. The interaction of bS1 protein with 23S rRNA has never been reported before, and is only observed in Mycobacteria because of its long rRNA extension H54a in the 23S rRNA. Because of the bS1-H54a interaction in the hibernating state 70S, H54a moves about 16.4 Å (about 15° angular tilt at A1564–G1605) towards mRNA exit site in the hibernating state when compared with the P/P state where H54a is strategically located in between the tRNA ejection and mRNA exit sites (Figs 4 and 5). This suggests a possible function of this Mycobacteria-specific H54a rRNA segment during both the translation elongation (involved in tRNA ejection) and initiation or resuscitation from hibernation (stabilizing bS1).

## Methods

### Isolation and purification of *M. smegmatis* ribosomes

*Ms* strain mc^2^ 155 was provided by Professor Gerhard Gruber (Nanyang Technological University, Singapore). *Ms* culture were grown in Luria-Bertani medium supplemented with 0.05% (v/v) Tween-80 and 0.2% (v/v) glycerol in shaker flasks (220 rpm, 37 °C). Cells were harvested at log phase (24 h) and stationary phase (48 h) for ribosome preparation by ultra-centrifugation (7,500 g, 20 min, 4 °C). Cell pellet was resuspended in extraction buffer (40 mM Hepes pH 7.5, 500 mM Potassium acetate, 25 mM Magnesium acetate, 250 mM Sucrose, and 5 mM β-mercaptoethanol) and lysed by sonication at 30% amplitude (10 secs ON and 20 secs OFF) for 5 min. The lysed cells were further broken down using microfluidizer and a clear lysate was obtained by centrifugation (15,000 g for 45 min). The supernatant was overlaid over sucrose cushion (40 mM Hepes pH 7.5, 500 mM Potassium acetate, 10 mM Magnesium acetate, 750 mM Sucrose, and 5 mM β-mercaptoethanol) for crude ribosome preparation by centrifugation (100,000 g for 2.5 h). The crude ribosome pellets were resuspended in the grid buffer (20 mM Hepes pH 7.5, 50 mM Potassium acetate, 10 mM Magnesium acetate, 5 mM β-mercaptoethanol, 0.1% PI pill/ml, and 1 unit/ml RNASin) over ice for about 2 h. Crude ribosomes were further purified over linear sucrose gradient of 10–40% (20 mM Hepes pH 7.5, 50 mM Potassium acetate, 10 mM Magnesium acetate, 5 mM β-mercaptoethanol, 0.1% PI pill/ml, 1 unit/ml RNASin, and 10/40% Sucrose) by centrifugation at 111,132 g for 4 h. After centrifugation, the ribosomes were fractionated on gradient station (Biocomp Instruments) at a speed of 0.34 cm/sec with a fraction size of 20 drops/tube. The 70S fractions were pooled together and overlaid on low salt sucrose cushion (20 mM Hepes pH 7.5, 50 mM Potassium acetate, 10 mM Magnesium acetate, 750 mM Sucrose, and 5 mM β-mercaptoethanol) and centrifuged at 100,000 g for 2.5 h. The purified ribosome pellets obtained after centrifugation were resuspended in the grid buffer over ice for about 2 h. The concentration of ribosome was measured using the spectrophotometer. Small aliquots were made and snap frozen in liquid nitrogen and stored at −80 °C until further use.

### Ribosome purification under different salt conditions

*Ms* strain mc^2^ 155 culture were grown as stated before and harvested at three different time intervals, 24, 48, and 66 h. Crude ribosomes were prepared under two different salt conditions, high salt (40 mM Hepes pH 7.5, 500 mM Potassium acetate, 25 mM Magnesium acetate, 250 mM Sucrose, and 5 mM β-mercaptoethanol) and low salt (40 mM Hepes pH 7.5, 50 mM Potassium acetate, 25 mM Magnesium acetate, 250 mM Sucrose, and 5 mM β-mercaptoethanol). Crude ribosomes were then purified over linear sucrose gradient of 10–40% (20 mM Hepes pH 7.5, 50 mM Potassium acetate, 10 mM Magnesium acetate, 5 mM β-mercaptoethanol, 0.1% PI pill/ml, 1 unit/ml RNASin, and 10/40% Sucrose) by centrifugation at 111,132 g for 4 h and fractionated with the gradient station (Biocomp Instruments) at a speed of 0.34 cm/sec. This experiment was performed to observe the formation of different forms of ribosomes (70S and/or 100S) under different growth period of *Ms*.

### Protein Identification by Mass spectrometry

70S ribosomes purified from log phase and stationary phase were analysed by SDS-PAGE. The protein identification of the band predicted to be bS1 based on its migration pattern on SDS-PAGE was carried out by MALDI-TOF/TOF. The peptide mass fingerprinting results were used in the Swiss-Prot database using the Mascot search engine to identify the protein.

### Electron Microscopy

For cryo-EM, quantifoil R2/2 carbon coated holey grids were used and glow discharged at 40 mA for 30 seconds prior to sample application. 3.5 µl of purified ribosome at the concentration of 180 nM were applied onto the glow-discharged grids. The grids were incubated with sample for 30 seconds in 100% humidity at 4 °C and blotted for 2.5 seconds before plunge-frozen into liquid ethane with FEI Vitrobot. Ribosomes purified from the log phase (70S-log) were imaged at FEI Titan Krios electron microscope (at National University of Singapore) operating at 300 kV. Images were recorded over 48 h on a FEI Falcon II direct electron detector at a calibrated magnification of 126,000 (yielding a pixel size of 1.11 Å). Defocus values for this dataset ranged from 0.5 to 3.5 μm. Images were acquired in movie mode using 23 movie frames (combined dose of 35 electrons per Å^2^). For the stationary phase purified ribosomes (70S-stat), images were collected on our in-house FEI Tecnai Arctica electron microscope (at Nanyang Technological University, Singapore) operating at 200 kV. 70S-stat images were recorded over 48 h on a FEI Falcon II direct electron detector at the calibrated magnification of 71,000 × (pixel size of 1.9 Å, defocus range 0.5 to 3.5 μm). Images were acquired in movie mode using 7 movie frames (combined dose of 26 electrons per Å^2^). In both datasets, images that showed significant drift or astigmatism based on their power spectra were manually discarded. The good images were corrected for beam-induced drift with Motioncorr^44^.

### Image processing

Particles were picked with EMAN 2.1 in a semi-automated mode^45^. Contrast transfer function (CTF) parameters were estimated with CTFFIND3^46^. RELION 1.4^47^ was used for image processing following the standard methods for processing of cryo-EM ribosome datasets^23,48^. A reference-free single round 2D classification was employed to discard the bad particles. The good particles were then subjected to various rounds of 3D classification to identify homogenous states with a filtered *Ec* SecA bound 70S ribosome map as a reference^49^. In 70S-log dataset, after performing two rounds of 3D classification and another additional round of 3D classification with fine angular sampling, the majority of the particles (>99%) were classified into one class yielding an average 3.6 Å resolution ribosome map. After including the movie frames in the 3D refinement, the resolution was further improved to 3.4 Å. This map was identified as P/P state 70S ribosome because of the presence of strong density for P-site tRNA in non-rotated 70S.

Focused mask refinement of LSU and SSU resulted in two maps at an average resolution of 3.2 Å and 3.7 Å, respectively. Local resolutions were calculated using ResMap^50^ (Supplementary Fig. S2). The 70S-log dataset also produced a minor class (0.6% particles) refined to an average 12.7 Å resolution (third round of 3D classification with fine angular sampling), which represents the trans-translating state of 70S ribosome with tmRNA bound at the P-site (Supplementary Fig. S1). The first 16 movie frames (combined dose of 24 electrons per Å^2^) were used for the final 3D reconstructions.

70S-stat Arctica dataset was processed in a similar manner with RELION 1.4. A total of 748 micrographs were selected for the processing of the 70S-stat dataset. EMAN 2.1 was used to pick a total of 114,282 particles. 91,303 particles grouped in a major class of 70S during 3D classification which was further resolved to an average resolution of 4.1 Å during refinement. All the 7 movie frames were used during processing. Analysis of this map showed that 70S exists in the hibernating state in this dataset, as evident by the additional density present at inter-subunit space for HPF. Resolutions for all the maps were calculated according to the gold standard Fourier Shell Correlation (FSC) = 0.143 criterion (Supplementary Figs S2 and S3)^51^. An illustration of data processing scheme for the 70S-log dataset is given in Supplementary Fig. S1. The high-resolution P/P state maps (3.4 Å for 70S, 3.2 Å for 50S, and 3.7 Å for 30S) were used for model building and refinement of r-proteins and rRNAs. The map quality of the P/P state and Hibernating state are compared in the Supplementary Fig. S15.

### Model building and refinement

*Ms* r-protein sequences were obtained from KEGG database for *Ms*^52^ (Supplementary Tables S1–S5). *Ec* r-proteins (PDB ID: 4YBB) were used as a template to generate homology models with I-TASSER for almost all of the r-proteins of *Ms* LSU and SSU^53^. *Ms* bL25 is more than double the size of *Ec* bL25 and to model this protein *Tth* bL25 was used as a template (PDB ID: 4V5D)^54^. bS22 and bL37 models were used from the recently published 70S ribosome structure of *Ms*^19^. Homology models were then rigid body fitted into the density maps obtained for LSU and SSU in UCSF Chimera^55^. Original maps (without post-processing procedure in RELION 1.4) were used for modelling the highly flexible parts of the map. Each protein was extensively rebuilt with side chains into the map density by applying Ramachandran restraints in COOT^56^. Ribosomal RNA sequences, 23S rRNA, 16S rRNA, and 5S rRNA were obtained from Pubmed^57^. rRNA sequence alignment and model building was done with *Ec* as the template in ModeRNA server^58^. rRNA models were fitted and rebuilt into density with UCSF Chimera and COOT, respectively. Extensions in r-proteins and rRNAs were built *de novo* in COOT. Ramachandran restraints were applied throughout in COOT. ‘Real Space Refine Zone’ and ‘Regularize Zone’ in COOT helped in manual model building and in obtaining optimal fitting. The fitting of LSU and SSU models were further improved with *phenix.real_space_refine*^59^ with RNA-base pairing restraints (generated by ‘PDB to 3D Restraints’ server) and secondary structure restraints for RNA and proteins, respectively. The LSU and SSU models were combined and rigid body docked into the 70S map. An *Ec* derived P-tRNA^fMet^ (PDB ID: 5AFI)^60^ was fitted onto the density of P-tRNA in the 70S map. The complete 70S model was further refined with *phenix.real_space_refine* to remove inter-subunit clashes. The P/P state of 70S model served both as a template and reference for modeling in the trans-translating and the hibernating state ribosome maps.

tmRNA sequence for *Ms* was obtained from tmRNA database^61^. *Tth* tmRNA (PDB ID: 3IYR)^12^ was used as template for tmRNA model building with ModRNA server. *Ms* tmRNA was rigid body docked into the 12.5 Å map of the trans-translating state of ribosome. *Tth* SmpB protein (PDB ID: 3IYR)^12^ was used as a template to build homology model for *Ms* SmpB protein in I-TASSER, and rigid body docked into the density for SmpB protein. An *Ec* derived A-tRNA^fMet^ (PDB ID: 5AFI)^60^ was rigid body docked onto the density of A-tRNA in the 70S map.

The 70S model obtained for the P/P state ribosome was used as a 70S model for hibernating state. HPF from *Sa* (PDB ID: 5ND8)^8^ was used as the template to generate homology model for *Ms* HPF with I-TASSER and rigid body docked into the density of HPF at the inter-subunit space. An *Ec* derived E-tRNA^fMet^ (PDB ID: 5AFI)^60^ was fitted onto the density of E-tRNA in the 70S map. *Ms* bS1 protein sequence was obtained from *Ms* KEGG database and a homology model was generated with Phyre2 software^62^. The best Phyre2 result was the model containing 216 residues of S1 protein from *Streptococcus pneumoniae* (PDB ID: 3GO5). *Ms* bS1 was built with unassigned UNK residues as the backbone and rigid body docked into the density observed for bS1 protein near the mRNA exit site. The fitting of the complete 70S model into hibernating state 70S ribosome map was further refined with *phenix.real_space_refine* by applying base-pairing and secondary structure restraints for RNA and proteins, respectively.

### Model Validation

Model validation for P/P state 70S ribosome model was done with MolProbity server^63^. To validate against model over-fitting, FSC curve between the final reconstructed cryo-EM maps and the maps generated from refined atomic coordinates were computed with resolution calculated according to the FSC = 0.5 criterion. The observed FSC curves and computed FSC curves display good agreement and thus showed the absence of any model-overfitting (Supplementary Fig. S2). To show the local agreement between map and models, maps were colored using ‘*vop localCorrelation*’ in UCSF Chimera (Supplementary Fig. S16)^55^. Model validation statistics for the P/P state 70S ribosome model are presented in Supplementary Table S6. The pixel size for both the maps were reassessed and the best fitting was achieved at pixel size 1.05 for 70S-log dataset maps and 1.28 for 70S-stat dataset map.

### Figure Generation

Figures were prepared in UCSF Chimera^55^.

### Accession Numbers

EM maps and atomic coordinates have been deposited with the Electron Microscopy Data Bank and Protein Data Bank under accession codes of EMD 6922 and PDB 5ZET for 50S-P/P state, EMD 6923 and PDB 5ZEU for 30S-P/P state, EMD 6920 and PDB 5ZEB for 70S-P/P state, EMD 6925 and PDB 5ZEY for 70S-trans-translating state, EMD 6921 and PDB 5ZEP for 70S-hibernating state, respectively.

## Electronic supplementary material

SUPPLEMENTARY_INFORMATION

## Data Availability

All data generated or analysed during this study are included in this published article (and its Supplementary Information files).
