# Supplementary material for: Structures of Mycobacterium smegmatis 70S ribosomes in complex with HPF, tmRNA, and P-tRNA
Source: Sci Rep. 2018 Sep 11;8:13587. doi: 10.1038/s41598-018-31850-3 (PMC6133939; doi:10.1038/s41598-018-31850-3)
Supplement: Supplementary file 1 — SUPPLEMENTARY_INFORMATION [file 41598_2018_31850_MOESM1_ESM.pdf]

SUPPLEMENTARY INFORMATION

for the following manuscript:

Structure of *Mycobacterium smegmatis* 70S ribosomes in complex with HPF, tmRNA and P-tRNA

Satabdi Mishra[1#], Tofayel Ahmed[1#], Anu Tyagi[1], Jian Shi[2] and Shashi Bhushan[1,3,*]

[1]School of Biological Sciences, Nanyang Technological University, Singapore
[2]Center for BioImaging Sciences, National University of Singapore, Singapore
[3]NTU Institute of Structural Biology, Nanyang Technological University, Singapore

[#] Equal contributions
* To whom correspondence should be addressed. Tel: +65 6592 3673; Fax: +65 6791 3856;
E-mail: sbhushan@ntu.edu.sg

Supplementary Table S1. Composition of P/P state SSU model.

**Protein/RNA name**	**Chain ID in SSU**	**Uniprot ID (GenBank ID)**	**Complete size**	**Modeled**	**Homologous *Ec* size**	**comments**
16S rRNA	a	X52922.1	1528	9-1518	1541	
uS2	b	A0QVB8	277	1-228	241	
uS3	c	A0QSD7	275	1-210	233	
uS4	d	A0QSL7	201	2-201	206	
uS5	e	A0QSG6	214	17-214	167	
bS6	f	A0A0D6J3X3	96	1-96	135	
uS7	g	A0QS97	156	1-156	179	
uS8	h	A0QSG3	132	3-132	130	
uS9	i	A0QSP9	150	25-150	130	
uS10	j	A0QSD0	101	5-101	103	
uS11	k	A0QSL6	138	22-138	129	
uS12	l	A0QS96	124	2-123	124	
uS13	m	A0QSL5	124	2-117	118	
uS14	n	A0QSG2	61	2-61	101	
uS15	o	A0QVQ3	89	2-88	89	
bS16	p	A0QV37	156	2-114	52	
uS17	q	A0QSE0	98	6-97	84	
bS18	r	A0R7F7	84	16-79	75	
uS19	s	A0QSD5	93	6-83	92	
bS20	t	A0R102	86	3-86	87	
bS22	u	A0QR10	33	2-33	-	
P-site $tRNA^{fMet}$	v	CP011124.1	77	1-77	77	*Ec* P-$tRNA^{fMet}$

Supplementary Table S2. Composition of P/P state LSU model.

Protein/RNA name	Chain ID in LSU	Uniprot ID (GenBank ID)	Complete size	Modeled	Homologous *Ec* size	comments
23S rRNA	A	NR_076104.1	3120	2-3119	2897	
5S rRNA	B	NR_075650.1	118	1-117	120	
uL1	-	A0QS46	235	-		not visible
uL2	C	A0QSD4	278	3-275	273	
uL3	D	A0QSD1	217	2-215	209	
uL4	E	A0QSD2	215	3-209	201	
uL5	F	A0QSG1	187	6-186	179	
uL6	G	A0QSG4	179	2-177	177	
bL7/bL12	-			-		not visible
bL9	H	A0R7F6	151	1-151	149	
uL10	I	A0QS62	175	1-126	165	
uL11	J	A0QS45	142	10-142	142	
uL13	K	A0QSP8	147	1-147	147	
uL14	L	A0QSF9	122	1-121	123	
uL15	M	A0QSG8	147	3-147	144	
uL16	N	A0QSD8	138	1-134	136	
bL17	O	A0QSL9	199	2-118	127	
uL18	P	A0QSG5	127	2-127	117	
bL19	Q	A0QV42	113	1-113	115	
bL20	R	A0QYU6	129	2-125	118	
bL21	S	A0R151	103	2-103	103	
uL22	T	A0QSD6	153	6-119	110	
uL23	U	A0QSD3	100	4-97	100	
uL24	V	A0QSG0	105	1-105	104	
bL25	W	A0R3D2	215	6-193	94	
bL27	X	A0R150	88	5-86	85	
bL28	Y	A0QV03	64	2-64	78	
uL29	Z	A0QSD9	77	5-57	63	
uL30	1	A0QSG7	61	2-61	59	
bL31	2	A0R215	82	1-66	70	
bL32	3	A0R3I9	57	2-55	57	
bL33	4	A0QS39	55	6-55	55	
bL34	5	A0R7K0	47	3-47	46	
bL35	6	A0QYU7	64	2-64	65	
bL36	7	A0QSL4	37	1-37	38	
bL37	8	A0QTP4	24	2-24	-	

Supplementary Table S3. Composition of hibernating state LSU model.

Protein/RNA name	Chain ID in 50S	Uniprot ID (GenBank ID)	Complete size	Modeled	Homologous *Ec* size	comments
23S rRNA	A	NR_076104.1	3120	2-3119	2897	
5S rRNA	B	NR_075650.1	118	1-117	120	
uL1	-	A0QS46	235	-		not visible
uL2	C	A0QSD4	278	3-275	273	
uL3	D	A0QSD1	217	2-215	209	
uL4	E	A0QSD2	215	3-209	201	
uL5	F	A0QSG1	187	6-186	179	
uL6	G	A0QSG4	179	2-177	177	
bL7/bL12	-			-		not visible
bL9	H	A0R7F6	151	1-151	149	
uL10	I	A0QS62	175	1-126	165	
uL11	J	A0QS45	142	10-142	142	
uL13	K	A0QSP8	147	1-147	147	
uL14	L	A0QSF9	122	1-121	123	
uL15	M	A0QSG8	147	3-147	144	
uL16	N	A0QSD8	138	1-134	136	
bL17	O	A0QSL9	199	2-118	127	
uL18	P	A0QSG5	127	2-127	117	
bL19	Q	A0QV42	113	1-113	115	
bL20	R	A0QYU6	129	2-125	118	
bL21	S	A0R151	103	2-103	103	
uL22	T	A0QSD6	153	6-119	110	
uL23	U	A0QSD3	100	4-97	100	
uL24	V	A0QSG0	105	1-105	104	
bL25	W	A0R3D2	215	6-193	94	
bL27	X	A0R150	88	5-86	85	
bL28	Y	A0QV03	64	2-64	78	
uL29	Z	A0QSD9	77	5-57	63	
uL30	v	A0QSG7	61	2-61	59	
bL31	y	A0R215	82	1-66	70	
bL32	z	A0R3I9	57	2-55	57	
bL33	1	A0QS39	55	6-55	55	
bL34	2	A0R7K0	47	3-47	46	
bL35	3	A0QYU7	64	2-64	65	
bL36	4	A0QSL4	37	1-37	38	
bL37	5	A0QTP4	24	2-24	-	

Supplementary Table S4. Composition of hibernating state SSU model.

**Protein/RNA name**	**Chain ID in SSU**	**Uniprot ID (GenBank ID)**	**Complete size**	**Modeled**	**Homologous *Ec* size**	**comments**
16S rRNA	a	X52922.1	1528	9-1518	1541	
uS2	b	A0QVB8	277	1-228	241	
uS3	c	A0QSD7	275	1-210	233	
uS4	d	A0QSL7	201	2-201	206	
uS5	e	A0QSG6	214	17-214	167	
bS6	f	A0A0D6J3X3	96	1-96	135	
uS7	g	A0QS97	156	1-156	179	
uS8	h	A0QSG3	132	3-132	130	
uS9	i	A0QSP9	150	25-150	130	
uS10	j	A0QSD0	101	5-101	103	
uS11	k	A0QSL6	138	22-138	129	
uS12	l	A0QS96	124	2-123	124	
uS13	m	A0QSL5	124	2-117	118	
uS14	n	A0QSG2	61	2-61	101	
uS15	o	A0QVQ3	89	2-88	89	
bS16	p	A0QV37	156	2-114	52	
uS17	q	A0QSE0	98	6-97	84	
bS18	r	A0R7F7	84	16-79	75	
uS19	s	A0QSD5	93	6-83	92	
bS20	t	A0R102	86	3-86	87	
bS22	u	A0QR10	33	2-33	-	
E-site $tRNA^{fMet}$	w	CP011124.1	77	1-77	77	*Ec* E-$tRNA^{fMet}$
HPF	x	A0QTK6	230	31-130	95	Rigid body fitted
bS1	0	A0QYY6	479	223 residues	556	Rigid body fitted

Supplementary Table S5. Composition of trans-translating state model.

Protein/RNA name	Chain ID in SSU	Uniprot ID (GenBank ID)	Complete size	Modeled	Homologous *Ec* size	comments
tmRNA	A	HG522090.1	369	1-369	363	Rigid body fitted
A-site $tRNA^{fMet}$	B	CP011124.1	77	1-77	77	*Ec* A-$tRNA^{fMet}$, Rigid body fitted
SmpB	C	A0QU63	161	1-161	160	Rigid body fitted

Supplementary Table S6. Model refinement statistics for the *Ms* 50S and 30S ribosome subunits and the complete 70S ribosome in P/P state.

Field	SSU	LSU	70S Ribosome
**Data Collection**			
Particles	391,837	391,837	391,837
Pixel Size (Å)	1.05	1.05	1.05
Defocus Range (µm)	0.3 – 4.3	0.3 – 4.3	0.3 – 4.3
Voltage (kV)	300	300	300
Electron Dose ($e^{-}Å^{-2}$)	35	35	35
**Model Composition**			
Non-hydrogen atoms	52,674	97,108	148,419
Protein residues	2,406	3,687	6,093
RNA bases	1,506	3,207	4,713
**Model Refinement**			
Resolution (Å)	3.28	3.7	3.4
Map sharpening B-factor ($Å^2$)	-134.86	-85.95	-84.62
CC_{map_model}	0.789	0.775	0.804
**RMS deviation**			
Bonds (Å)	0.02	0	0.01
Angles (°)	1.04	0.71	1.03
**Validation (proteins)**			
Molprobity score	2.22 (63rd percentile)	2.64 (39th percentile)	2.57 (43rd percentile)
Clashscore, all atoms	16.04 (45th percentile)	16.47 (43rd percentile)	18.45 (36th percentile)
Poor rotamers (%)	0.90	3.4	2.39
Favoured rotamers (%)	94.05	90.42	91.89
**Ramachandran plot**			
Favoured (%)	91.08	90.91	90.98
Outliers (%)	0.42	1.13	0.85
**Validation (RNA)**			
Correct sugar puckers (%)	98.8	99.35	99.77
Good backbone conformations (%)	74.04	75.05	74.72
Bond Outliners (%)	0	0	0
Angle Outliners (%)	0.02	0.01	0.01

Figure S1.

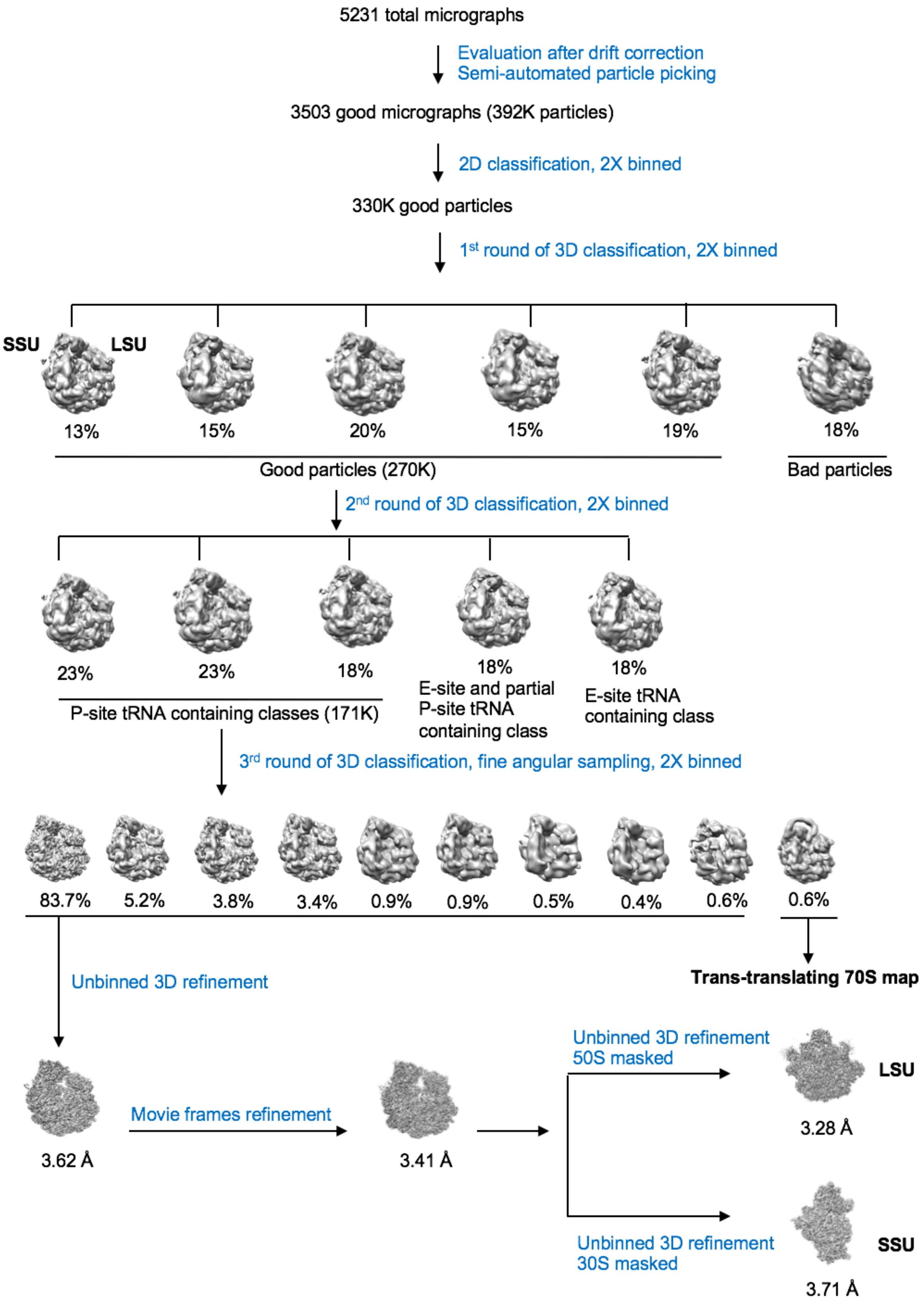
5231 total micrographs
Evaluation after drift correction
Semi-automated particle picking
3503 good micrographs (392K particles)
2D classification, 2X binned
330K good particles
1st round of 3D classification, 2X binned
SSU
LSU
13%
15%
20%
15%
19%
18%
Good particles (270K)
Bad particles
2nd round of 3D classification, 2X binned
23%
23%
18%
18%
E-site and partial P-site tRNA containing class
18%
E-site tRNA containing class
P-site tRNA containing classes (171K)
3rd round of 3D classification, fine angular sampling, 2X binned
83.7%
5.2%
3.8%
3.4%
0.9%
0.9%
0.5%
0.4%
0.6%
0.6%
Unbinned 3D refinement
Trans-translating 70S map
3.62 Å
Movie frames refinement
3.41 Å
Unbinned 3D refinement
50S masked
LSU
3.28 Å
Unbinned 3D refinement
30S masked
SSU
3.71 Å

Figure S1: Cryo-EM data processing pipeline for the 70S-log dataset. Out of 5231 collected micrographs, 3503 micrographs were selected as good micrographs showing minimal drift and no astigmatism. 392K particles were picked semi-automatically using EMAN 2.1[1]. Images were processed further in RELION 1.4[2]. 2X-binned particles were sorted with 2D classification. 330K good particles were selected and subjected to several rounds of 3D classification with an empty *Ec* 70S ribosome as reference[3]. In the 3rd round of 3D classification, fine angular sampling was applied and one 3D class (with 1313 particles, 0.6% particles) was identified as the trans-translating state of ribosome. The final resolution obtained for this class after un-binned 3D refinement was 12.5 Å. The rest of the un-binned particles were 3D-refined generating a 70S map at 3.62 Å resolution. Movie frames were then included in the 3D refinement, which yielded in a 3.41 Å resolved map for the 70S ribosome. Focused mask refinement of LSU and SSU resulted in two maps resolved to 3.28 and 3.71 Å, respectively.

Figure S2.

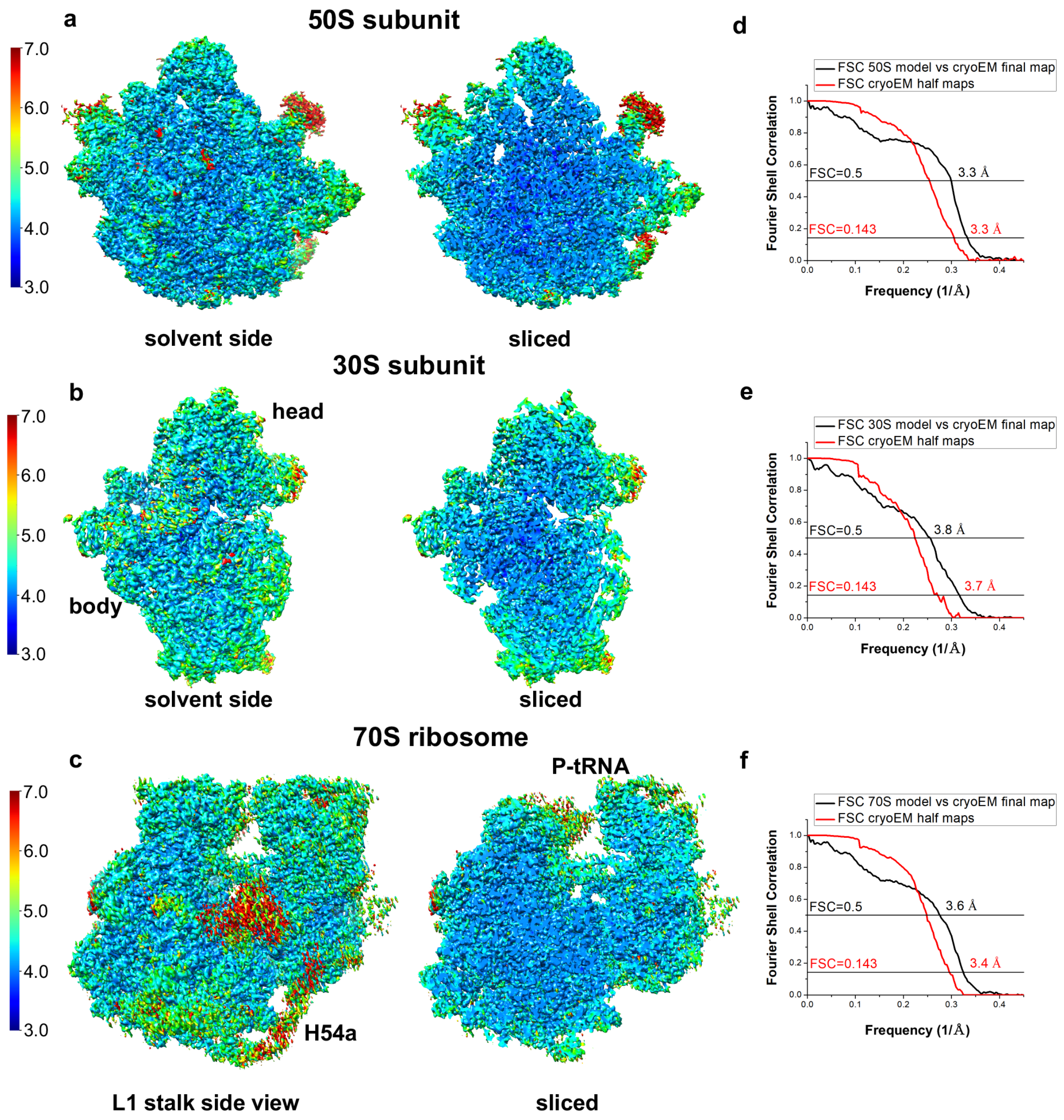

Figure S2: Estimation of resolution and validation against model-overfitting. (a-c) Local resolution estimation with ResMap[4] for the 50S and 30S subunits, and 70S ribosome. Both solvent side views (left) and sliced views (right) are shown. The core regions across all three maps are well-resolved (sliced view) compared to the peripheral regions. (**d-f)** Average resolution of the final refined Cryo-EM maps and validation against model-overfitting. Red: Fourier Shell Correlation (FSC) curves for 50S, 30S and 70S maps with estimated average resolutions of 3.3, 3.7, and 3.4 Å, respectively, calculated according to the FSC = 0.143 criterion[5]. Black: FSC curve computed between the final Cryo-EM maps and maps generated from refined atomic coordinates with resolution calculated according to the FSC = 0.5 criterion.

Figure S3.

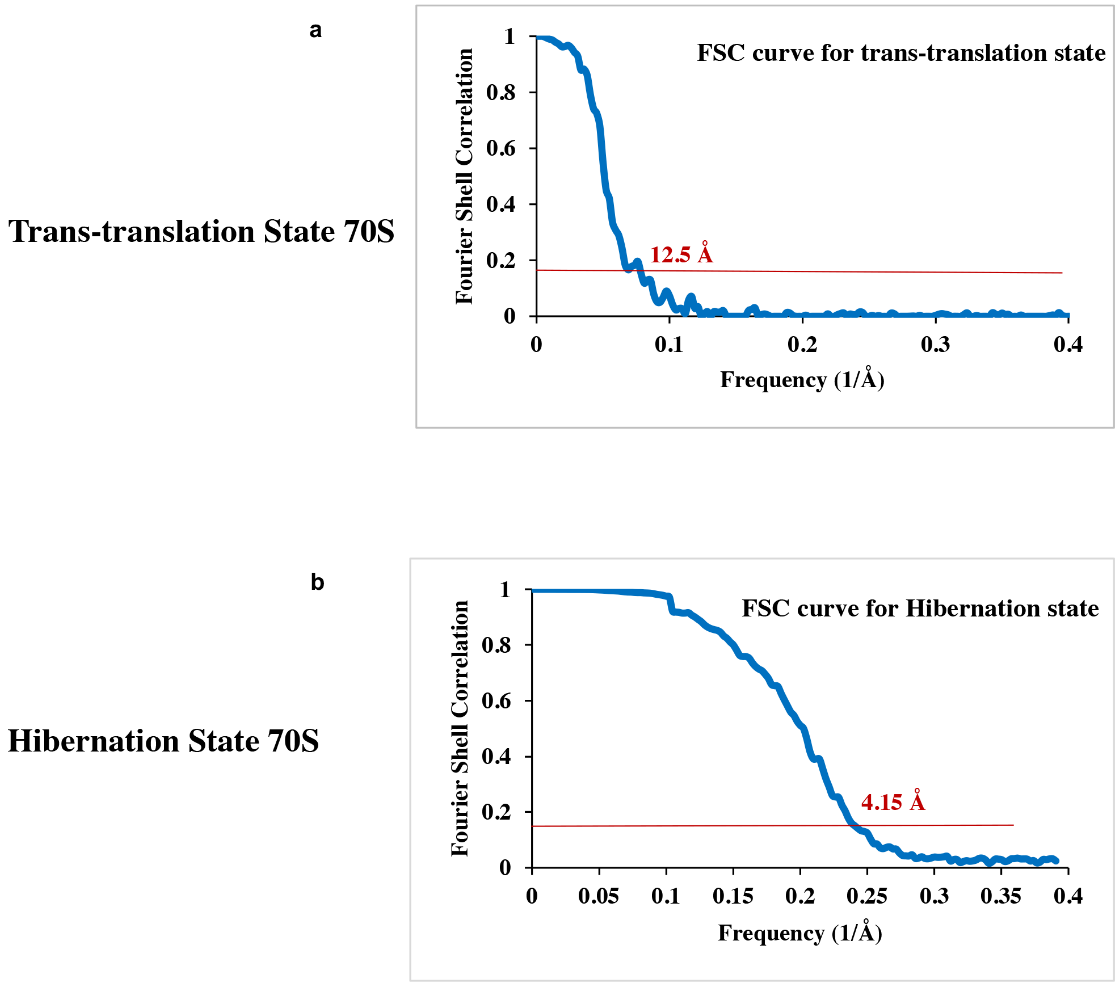

Figure S3: Resolution estimation for the trans-translation and hibernation states 70S ribosome maps. FSC curves for **(a)** trans-translation and **(b)** hibernation state 70S ribosome map as indicated, using FSC= 0.143 criterion[5].

Figure S4.

```
Ms        MSSHSMDSSRTMVVEDDRESTAGTTPERPHAEVVVKGRNVEVPDHFRTYVSEKLSRLERF   60
Sa        -----------------------------MIRFEIHGDNLTITDAIRNYIEEKIGKLERY   31
Tth       -----------------------------MNIYKLIGRNLEITDAIRDYVEKKLARLDRY   31
                                            : * *: : * :* *:.:*:.:*:*:

Ms        DKTIYLFDVELDHERNRRQRKNCQHVEITARGRGPVVRGEACADSFYTAFESAVQKLEGR   120
Sa        FNDVPNAVAHVKVKT---YSNSATKIEVTIPLKNVTLRAEERNDDLYAGIDLINNKLERQ   88
Tth       QDGELMAKVVLSLAGSP-HVEKKARAEIQVDLPGGLVRVEEEDADLYAAIDRAVDRLETQ   90
           .      . :.        :.  : *:     .  :* *    .:*:.::   ::** :

Ms        LRRAKDRRKIHYGD----KTPVSLAEATAKDPLAGLDVSREDGEARYNDGVAEHEPGRIV   176
Sa        VRKYKTRVNRKSRD--RGDQEVFVAELQE------M------QETPVDNETYDDNEIEII   134
Tth       VKRFRERRYVGKRHSYQGPPPPEVRDLEA------L------------RKPEEEEGPRIV   132
          ::: : *      .         : :         :                :.:  .*:

Ms        RIKDHPATPMTVDDALYEMELVGHDFFLFHDKETDRPSVVYRRHAFDYGLIRLA--     230
Sa        RSKEFSLKPMDSEEAVLQMNLLGHDFFVFTDRETDGTSIVYRRKDGKYGLIQTSEQ     190
Tth       RVKRFEMKPMDPEEAAFQMEALGHSFFVFRNAKTDEINVIYRRKDGNYGLIEPA--     186
          * * .  .**  ::*  :*: :**.**:* : :**  .::***:  .****. :
```

Figure S4: Sequence alignment of HPF from *Ms*, *Sa* and *Tth*. The sequence alignment between HPF from *M. smegmatis*, *S. aureus* and *T. thermophilus*. The sequence similarity between *Ms* and *Sa* is 43.5% and between *Ms* and *Tth* is 40.2%

Figure S5.

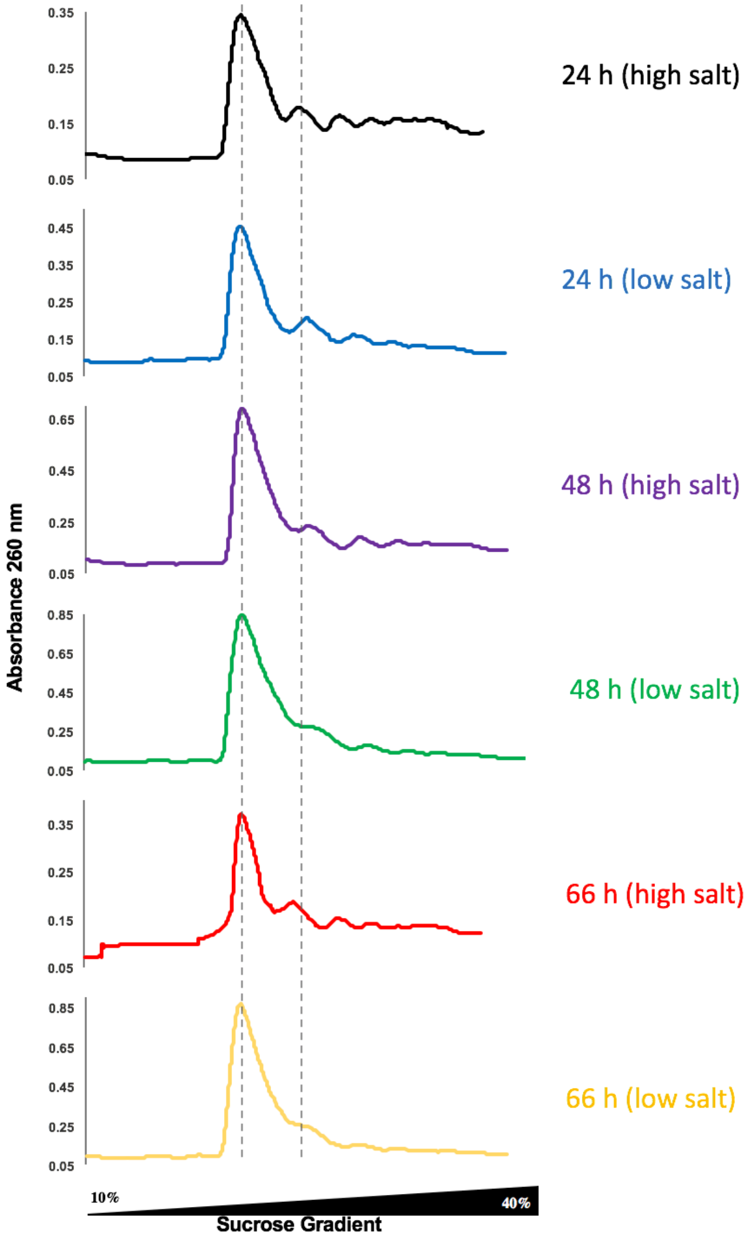

Figure S5: Ribosome purification under high and low salt conditions for *Ms* cultures harvested at different growth periods. Ribosomes purified from *Ms* culture harvested at different growth periods of 24, 48, and 66 h, as indicated. For each growth period, ribosomes were purified under low (50 mM Potassium acetate) and high salt (500 mM Potassium acetate) conditions. Crude ribosomes were then analysed by linear sucrose gradient (10 - 40%) to observe ribosomal profile under these conditions. Neither any of the ribosomal subunits (30S and 50S) nor the 100S ribosomal dimers could be detected under these conditions even after extended stationary phase (66 h growth) while 70S seems to be stably present during stationary phases.

Figure S6.

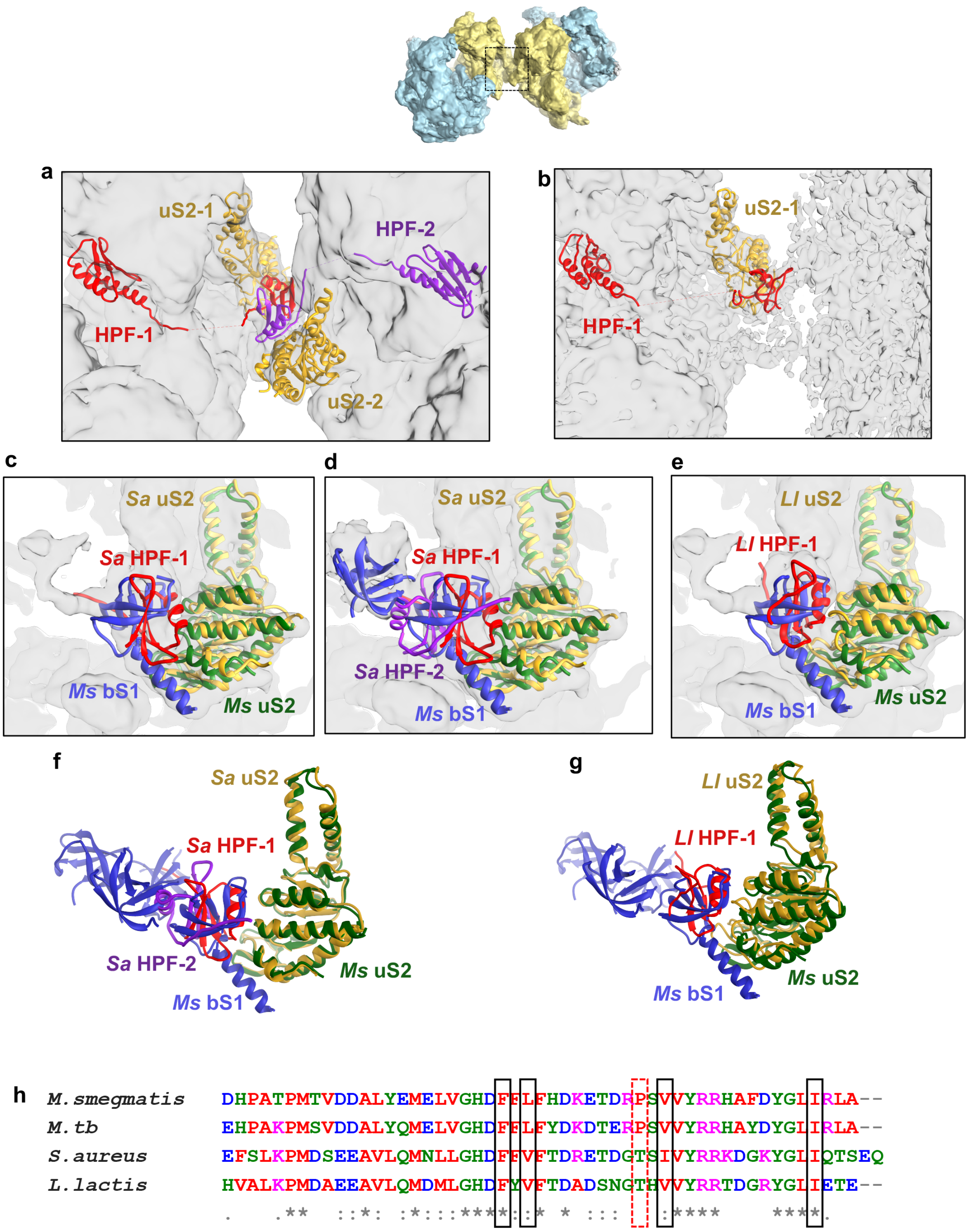
a
uS2-1
HPF-2
HPF-1
uS2-2
b
uS2-1
HPF-1
c
Sa uS2
Sa HPF-1
Ms bS1
Ms uS2
d
Sa uS2
Sa HPF-1
Sa HPF-2
Ms bS1
e
Ll uS2
Ll HPF-1
Ms bS1
Ms uS2
f
Sa uS2
Sa HPF-1
Sa HPF-2
Ms bS1
Ms uS2
g
Ll uS2
Ll HPF-1
Ms bS1
Ms uS2
h
M.smegmatis DHPATPMTVDDALYEMELVGHDFFLFHDKETDRPSVVYRRHAFDYGLIRLA--
M.tb EHPAKPMSVDDALYQMELVGHDFFLFYDKDTERPSVVYRRHAYDYGLIRLA--
S.aureus EFSLKPMDSEEAVLQMNLLGHDFFVFTDRETDGTSIVYRRKDGKYGLIQTSEQ
L.lactis HVALKPMDAEEAVLQMDMLGHDFYVFTDADSNGTHVVYRRTDGRYGLIETE--
.   .** ::*: :*:::*****:* * :::   :****   ****.

Figure S6: Comparison between *Ms* HPF, *Sa* HPF and *Ll* HPF. (a) Ribosome dimer from *Sa* (PDB: 5NG8) showing the HPFs from the two small subunits interacting with uS2 protein to form stable dimer[6]. CTD-HPF-1 (red) from SSU-1 interact with CTD-HPF-2 (purple) of SSU-2 of ribosome dimer. **(b)** Ribosome dimer from *Ll* (PDB: 5MYJ) showing the CTD-HPF-1 (red) and uS2-1 (yellow) protein at the dimer interface[7]. **(c)** Super-position of *Sa* CTD-HPF-1 (red) and *Sa* uS2-1 (yellow) with *Ms* bS1 OB-domain I (blue) and *Ms* uS2 (dark green), OB domain-I of *Ms* bS1 overlaps with the *Sa* CTD-HPF-1. **(d)** OB domains-I and II overlap the *Sa* CTD-HPF-2 (purple). **(e)** Super-position of *Ll* CTD-HPF (red) and *Ll* uS2 (yellow) with OB domain-I of *Ms* bS1 (blue) and *Ms* uS2 (dark green). **(f)** Super-position of *Ms* bS1 (blue) and *Ms* uS2 (dark green) with *Sa* CTD-HPF-1 (red), *Sa* CTD-HPF-2 (purple) and *Sa* uS2 (yellow). **(g)** Super-position of *Ms* bS1 (blue) and *Ms* uS2 (dark green) with *Ll* CTD-HPF (red) and *Ll* uS2 (yellow) **(c-g)** show that *Ms* bS1 sterically clashes with the interaction of CTD of HPF from *Sa* and *Ll* in their ribosome dimers. **(h)** sequence comparison of CTD between *Ms (M. smegmatis), Mtb (M. tuberculosis), Sa (S. aureus)* and *Ll (L. lactis).* One out of five conserved residues involved in HPF dimer interaction is not conserved in *Ms* and *Mtb* (shown in red dotted box)[7].

Figure S7.

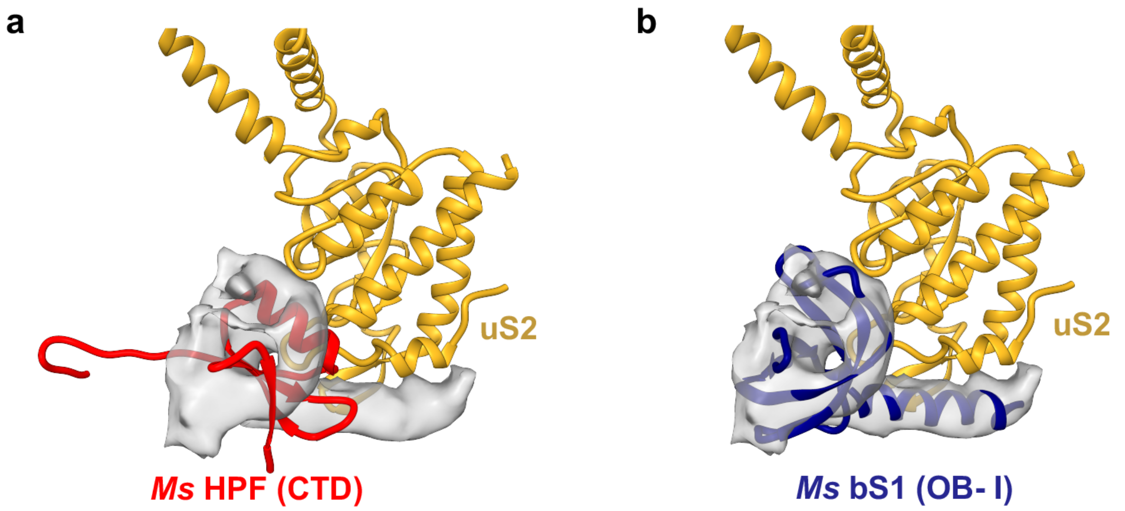

Figure S7. Analysis of the extra density near the mRNA exit channel in the Hibernating state map. The models of *Ms* HPF CTD **(a)** and *Ms* bS1 OB-I **(b)** were fitted into the extra density observed near the mRNA exit site of the hibernating state 70S map. The *Ms* bS1 fitted well into this density compared to *Ms* HPF CTD. The *Ms* HPF CTD is shown in the same orientation as found in the *S. aureus* and *L. lactis* 100S dimers[6,7].

Figure S8.

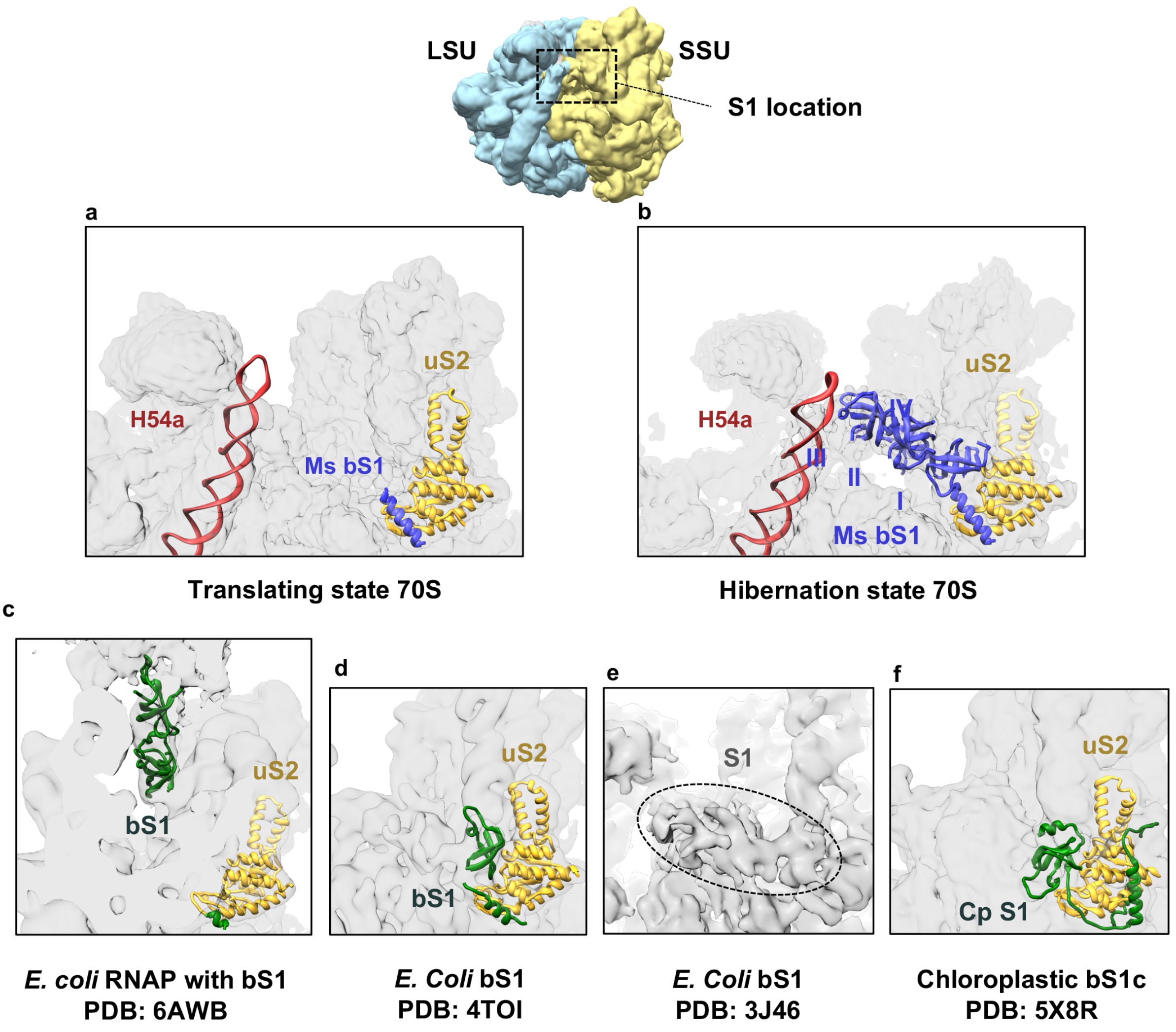

Figure S8: Comparison of S1 structure in association with the ribosome as captured in different studies. (a) *Ms* bS1 N-terminal α-helix (blue) in P/P map (filtered to 10 Å), along with H54a (deep red) and uS2 (yellow). **(b)** *Ms* bS1 (deep-blue) with its four OB domains in hibernating map along with H54a (deep red) and uS2 protein (yellow). **(c)** Cryo-EM density for bS1 in RNA polymerase-bound *Ec* SSU (PDB ID: 6AWB)[8]. Model for bS1 is shown in dark green and uS2 in yellow. **(d)** Cryo-EM density of bS1 in *Ec* SSU (PDB ID: 4TOI)[9]. **(e)** Cryo-EM bS1 density on the *Ec* ribosome bound to translocon SecY (PDB ID: 3J46)[10]. **(f)** Cryo-EM density of bS1c in the chloroplast SSU (PDB ID: 5X8R)[11].

Figure S9.

Matched peptides shown in **Bold Red**

```
  1 MPSPSVTSPQ VAVNDIGSAE DFLAAIDKTI KYFNDGDIVE GTIVKVDRDE
 51 VLLDIGYKTE GVIPSRELSI KHDVDPNEVV SVGDEVEALV LTKEDKEGRL
101 ILSKKRAQYE RAWGTIEELK EKDEAVKGTV IEVVKGGLIL DIGLRGFLPA
151 SLVEMRRVRD LQPYIGKEIE AKIIELDKNR NNVVLSRRAW LEQTQSEVRS
201 EFLNQLQKGA IRKGVVSSIV NFGAFVDLGG VDGLVHVSEL SWKHIDHPSE
251 VVQVGDEVTV EVLDVDMDRE RVSLSLKATQ EDPWRHFART HAIGQIVPGK
301 VTKLVPFGAF VRVEEGIEGL VHISELSERH VEVPDQVVQV GDDAMVKVID
351 IDLERRRISL SLKQANEDYT EEFDPSKYGM ADSYDEQGNY IFPEGFDPET
401 NEWLEGFDKQ REEWEARYAE AERRHKMHTA QMEKFAAAEA EAANAPVSNG
451 SSRSEESSGG TLASDAQLAA LREKLAGNA
```

Matched (bold red) stretches: 1–49 (MPSPSVTSPQ … LTKEDKEGR), 112–127 (AWGTIEELK EKDEAVK), 136–150 (GGLIL DIGLRGFLPA), 151–208 (SLVEMRRVRD … EFLNQLQK), 244–270 (HIDHPSE VVQVGDEVTV EVLDVDMDRE), 278–300 (ATQ EDPWRHFART HAIGQIVPGK), 301–377 (VTKLVPFGAF … EEFDPSK), 418–424 (YAE AERR), 435–474 (FAAAEA … LREK).

Gel Idx/Pos	273/L1	Instr./Gel Origin	ak072/180404	Process Status	Analysis Succeeded
Plate [#] Name	[1] mw2	Instrument Sample Name		Spectra	11

Rank	Protein Name	Species	Accession No.	Protein MW	Protein Score	Protein Score C. I. %	Total Ion Score	Total Ion C. I. %	Confirmed
1	30S ribosomal protein S1 OS=Mycobacterium smegmatis (strain ATCC 700084 / mc(2)155) GN=rpsA PE=1 SV		sp|A0QYY6|RS1_ MYCS2	53283.3	941	100	617	100	.T.

sequence coverage = 75%

Figure S9: Mass spectrometry analysis of bS1 protein from stationary phase 70S ribosomes. The sequences colored in bold red are the matched peptides from peptide mass fingerprinting. The sequence coverage obtained was 75%, with the highest hit for bS1 protein from *Mycobacterium smegmatis*.

Figure S10.

```
Ms_bS1          MPSPSVTSPQVAVNDIGSAEDFLAAIDKTIKYFNDGDIVEGTIVKVDRDEVLL-DIGYKT     59
Sa_bS1          -----------------MTE-EFN--ESMINDIKEGDKVTGEVQQVEDKQVVVHINGGKF     40
Ll_bS1          ------------------MN-EFETLLNSVEDVKVRDVVKGEILSVENGQATVAIVGTGV     41
                                   :  :    . :: .:  * * * : .*:  :. :   *

Ms_bS1          EGVIPSRELSIKHDVDPNEVVSVGDEVEALVLTK-----EDKEGRLILSKKRAQYERAWG     114
Sa_bS1          NGIIPISQLSTHHIDSPSEVVKEGDEVEAYVTKVEFD-EENETGAYILSRRQLETEKSYS     99
Ll_bS1          EGVLTLREITNDKDADINTFVKPGDVLDLLVIKQIVGKEAEGANVYLLSLKRLEARKAWT     101
                :*::   ::: .:  . . .*. ** ::  * .       :  .  :** :: : .:::

Ms_bS1          TIEELKEKDEAVKGTVIEVVKGGLILDI-GLRGFLPASLVEMRRVRDLQPYIGKEIEAKI     173
Sa_bS1          YLQEKLDNNEIIEAKVTEVVKGGLVVDV-GQRGFVPASLISTDFIEDFSVFDGQTIRIKV     158
Ll_bS1          QLEG--KEGEIVTVKVTKDVKGGLSVDYNGVRGFIPASMIDTYFVKDTKKFVGEEIEAKI     159
                 ::   .:.* :  .* : ***** :*  * ***:***::.   :.* . : *: *. *:

Ms_bS1          IELDKNRNNVVLSRRAWLEQTQSEVRSEFLNQLQKGAIRKGVVSSIVNFGAFVDLGGVDG     233
Sa_bS1          EELDPENNRVILSRKAVEQEENDAKKDQLLQSLNEGDVIDGKVARLTQFGAFIDIGGVDG     218
Ll_bS1          IEVNASENRFILSRRAVVEAEAIEMRKEAFAQLQEGDIVEGTVSRVTNFGAFVDLGGIDG     219
                 *:: ..*..:***:*  :      :.: : .*::* : .* *: :.:****:*:**:**

Ms_bS1          LVHVSELSWKHIDHPSEVVQVGDEVTVEVLDVDMDRERVSLSLKATQEDPWRHFARTHAI     293
Sa_bS1          LVHVSELSHEHVQTPEEVVSIGQDVKVKIKSIDRDTERISLSIKDTLPTPFENIKGQFHE     278
Ll_bS1          LVHVSELSHSRIKRPSDAVKPGDVVNVKILKLDPEAGRLSLSLKATQPGPWEQVEEKAPV     279
                ******** .::. *.:.*. *: *.*:: .:* :  *:***:* *   *:.:.

Ms_bS1          GQIVPGKVTKLVPFGAFVRVEEGIEGLVHISELSERHVEVPDQVVQVGDDAMVKVIDIDL     353
Sa_bS1          NDVIEGVVVRLANFGAFVEIAPGVQGLVHISEIAHKHIGTPGEVLEPGQQVNVKILGIDE     338
Ll_bS1          GSTVEGTVKRLTDFGAFVELYPGVEGLVHVSQISWERVENPKDVLKVGQVVNVKVLDVKP     339
                .. : * * :*. *****.:  *::****:*::: .::  * :*:: *: . **::.:.

Ms_bS1          ERRRISLSLKQANEDYTEEFDPSK-YG-------MADSYDEQGNYIFPEGFDPETNEWLE     405
Sa_bS1          ENERVSLSIKATLPNEDVVES--D---------PS-----TTKAYLENEEEDNPTIGDMI     382
Ll_bS1          AEERISLSIKALEEAPARPARNNDNDGEKRDRKPRAPRKAAKPSYDLPETQEGFSLADFL     399
                 ..*:***:*             .                    *   *  :  :   :

Ms_bS1          GFDKQREEWEARYAEAERRHKMHTAQMEKFAAAEAEAANAPVSNGSSRSEESSGGTLASD     465
Sa_bS1          GDKLKNLKL---------------------------------------------------     391
Ll_bS1          GEDFDINDL---------------------------------------------------     408
                * . .  .

Ms_bS1          AQLAALREKLAGNA      479
Sa_bS1          --------------      391
Ll_bS1          --------------      408
```

Figure S10: Sequence alignment of bS1 from *Ms*, *Sa* and *Ll*. The sequence alignment between bS1 from *M. smegmatis (Ms)*, *S. aureus (Sa)* and *L. lactis (Ll)* shows that *Ms* bS1 is significantly different when compared with *Sa* and *Ll* bS1.

Figure S11.

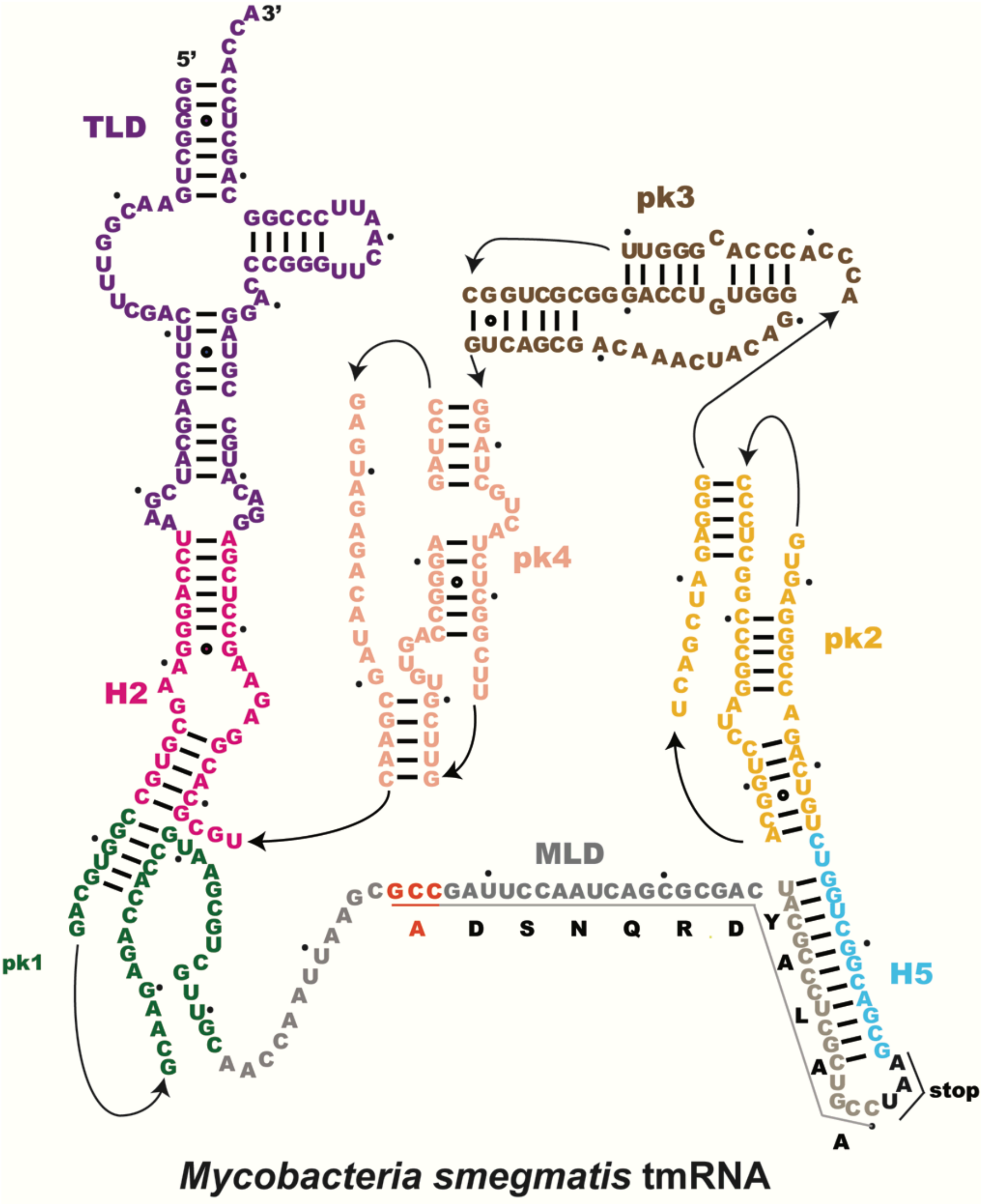

Figure S11: Secondary structure diagram of *Ms* tmRNA. Secondary structure of tmRNA from *Ms*, highlighting the structural domains TLD, MLD, pseudoknots and helices. MLD coding for protein degradation tag (ADSNQRDYALAA) is underlined with resume codon shown in red and stop codon in black. The different domains are colored similar to fig. 3b. The dots denote counts after every 10 nucleotides. Watson-crick base pairs are shown in lines (-), G◉U base pairs with rings (◉), and non-standard base pairs with dots (•). The figure is adapted from tmRNA database website[12].

Figure S12.

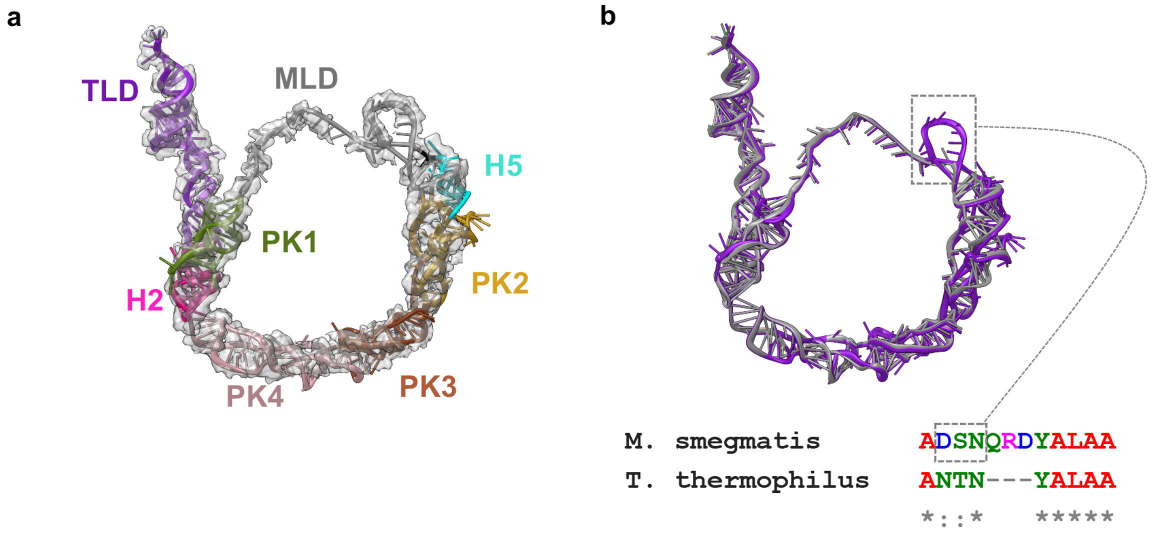

Figure S12: Structure of tmRNA in the trans-translating 70S map. (a) Fitted tmRNA model with the extracted density filtered to 15 Å. tmRNA density is shown in transparent grey surface and different domains of tmRNA are colored similar to fig. 3b. **(b)** Comparison of tmRNA from *Ms* (purple) and *Tth* (grey) (PDB: 3IYR) and sequence comparison of the degradation tags encoded by MLD. An insertion of 9 nucleotides is boxed in the MLD of *Ms*[13].

Figure S13.

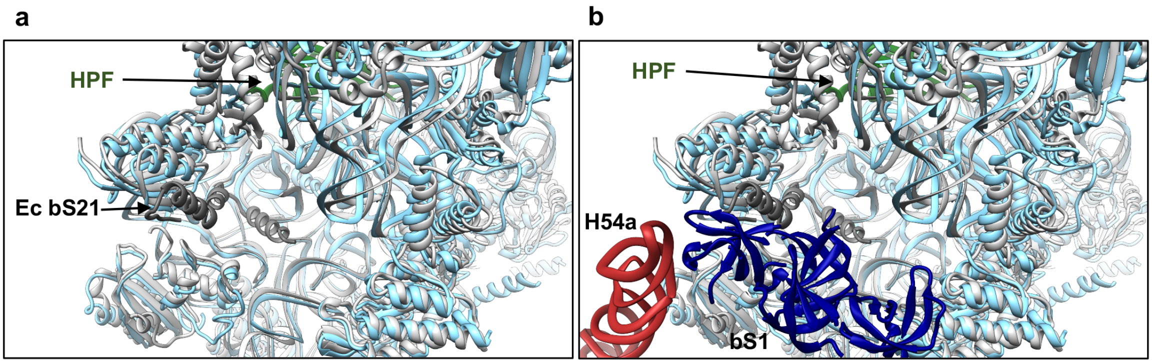

Figure S13: mRNA exit site. **(a)** Atomic models of the *Ms* (light blue) and *Ec* ribosomes (grey, PDB ID: 4YBB)[14] are super-positioned to show the overall conserved architecture of the mRNA exit site. Protein bS21 (highlighted in deep-grey) is absent in *Ms*. Models for the helix H54a and protein bS1 are hidden for clarity. **(b)** The super-position between *Ms* and *Ec* ribosome models as in (a) but with the presence of *Ms* bS1 (deep-blue) and *Ms* H54a (deep-red).

Figure S14.

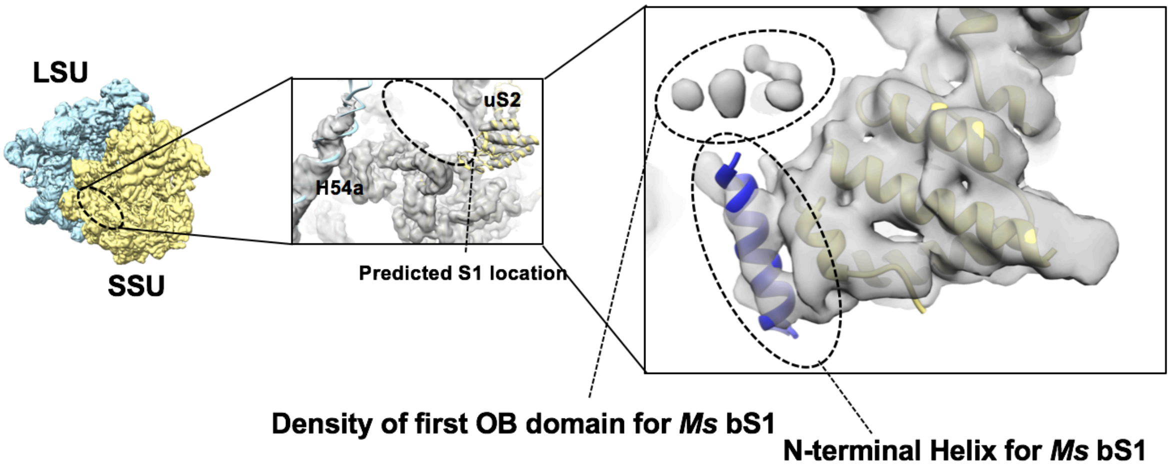

Figure S14: bS1 density in the P/P state 70S ribosome. bS1 density in P/P state 70S map when filtered to 10 Å and shown at 0.01 threshold in UCSF Chimera[15]. The density of N-terminal α-helix appears at this resolution. A fragmented density is observed for the first OB domain.

Figure S15.

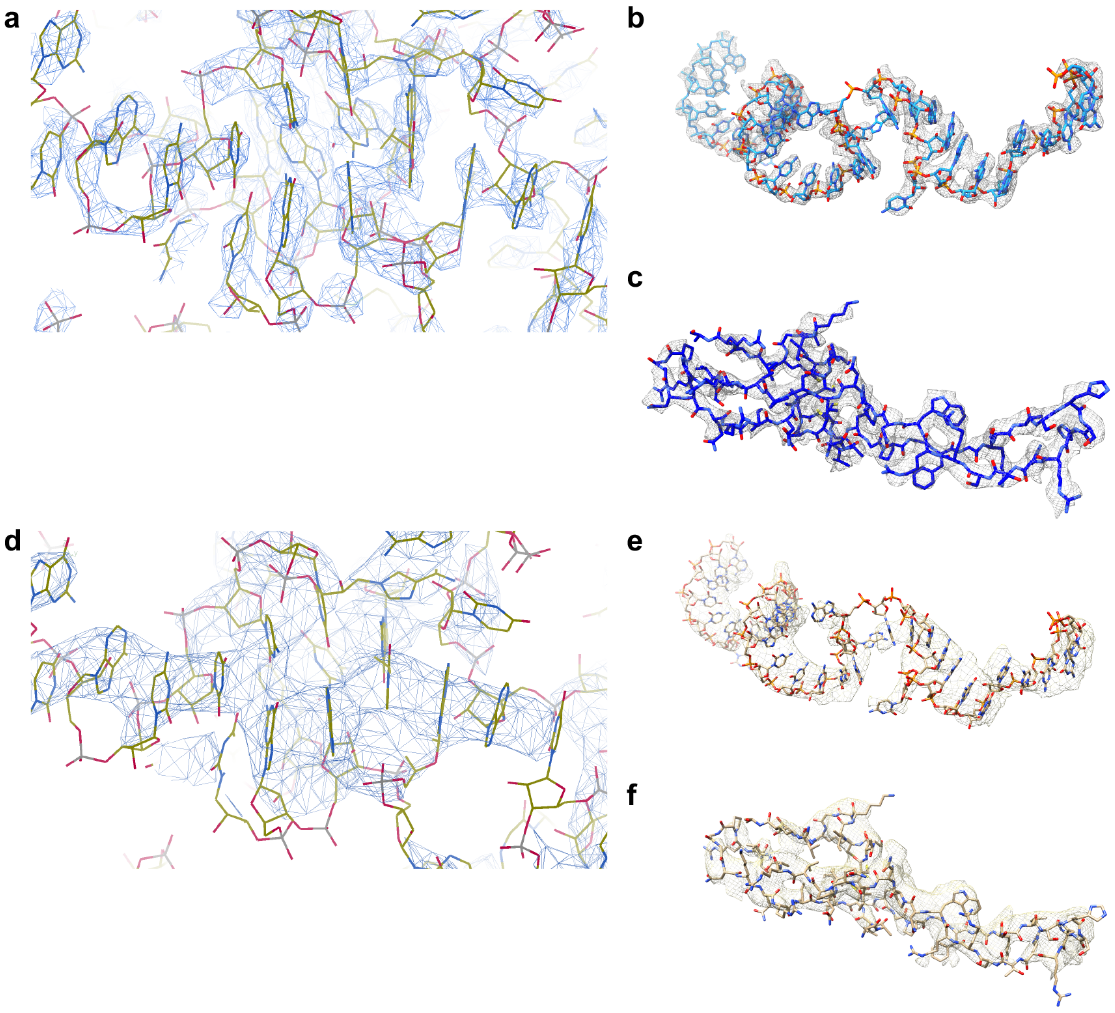

Figure S15: Quality of the P/P state (3.4 Å) and hibernating state (4.1 Å) maps. (a and d) A part of 23S rRNA (Helix 11) showing the quality of map and fitting of nucleotides. **(b and e)** Density and fitting of a small part of 23S rRNA (C2271-A2305) extracted in chimera. **(c and f)** Density and fitting of LSU protein bL28 extracted in chimera. **a-c**, P/P state (3.4 Å) map; **d-f** hibernating state (4.1 Å) map.

Figure S16.

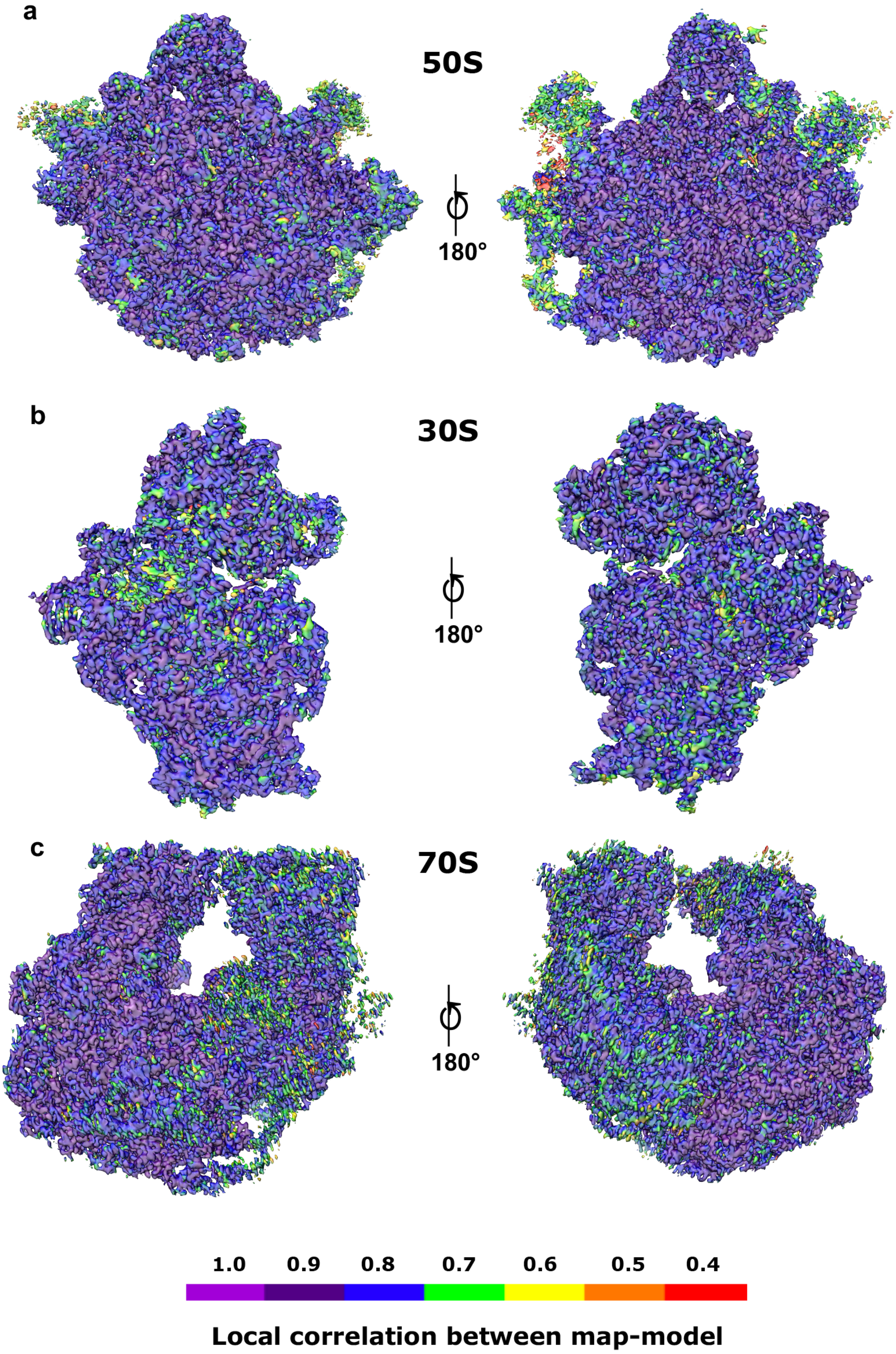
a
50S
180°
b
30S
180°
c
70S
180°
1.0
0.9
0.8
0.7
0.6
0.5
0.4
Local correlation between map-model

Figure S16: Local agreement between the cryo-EM maps and models. The 50S subunit **(a)**, 30S subunit **(b)**, and 70S **(c)** cryo-EM maps from P/P state are colored based on the local correlation values between the cryo-EM maps and respective atomic models (calculated using UCSF Chimera[15]) to indicate the presence of minimal overfitting in the final models.

References

1 Bell, J. M., Chen, M., Baldwin, P. R. & Ludtke, S. J. High resolution single particle refinement in EMAN2.1. *Methods* **100**, 25-34, doi:10.1016/j.ymeth.2016.02.018 (2016).

2 Kimanius, D., Forsberg, B. O., Scheres, S. H. & Lindahl, E. Accelerated cryo-EM structure determination with parallelisation using GPUs in RELION-2. *Elife* **5**, doi:10.7554/eLife.18722 (2016).

3 Fischer, N. *et al.* Structure of the E. coli ribosome-EF-Tu complex at <3 A resolution by Cs-corrected cryo-EM. *Nature* **520**, 567-570, doi:10.1038/nature14275 (2015).

4 Kucukelbir, A., Sigworth, F. J. & Tagare, H. D. Quantifying the local resolution of cryo-EM density maps. *Nat Methods* **11**, 63-65, doi:10.1038/nmeth.2727 (2014).

5 Rosenthal, P. B. & Henderson, R. Optimal determination of particle orientation, absolute hand, and contrast loss in single-particle electron cryomicroscopy. *J Mol Biol* **333**, 721-745 (2003).

6 Matzov, D. *et al.* The cryo-EM structure of hibernating 100S ribosome dimer from pathogenic Staphylococcus aureus. *Nat Commun* **8**, 723, doi:10.1038/s41467-017-00753-8 (2017).

7 Franken, L. E. *et al.* A general mechanism of ribosome dimerization revealed by single-particle cryo-electron microscopy. *Nat Commun* **8**, 722, doi:10.1038/s41467-017-00718-x (2017).

8 Demo, G. *et al.* Structure of RNA polymerase bound to ribosomal 30S subunit. *Elife* **6**, doi:10.7554/eLife.28560 (2017).

9 Byrgazov, K. *et al.* Structural basis for the interaction of protein S1 with the Escherichia coli ribosome. *Nucleic Acids Res* **43**, 661-673, doi:10.1093/nar/gku1314 (2015).

10 Park, E. *et al.* Structure of the SecY channel during initiation of protein translocation. *Nature* **506**, 102-106, doi:10.1038/nature12720 (2014).

11 Ahmed, T., Shi, J. & Bhushan, S. Unique localization of the plastid-specific ribosomal proteins in the chloroplast ribosome small subunit provides mechanistic insights into the chloroplastic translation. *Nucleic Acids Res* **45**, 8581-8595, doi:10.1093/nar/gkx499 (2017).

12 Hudson, C. M. & Williams, K. P. The tmRNA website. *Nucleic Acids Res* **43**, D138-140, doi:10.1093/nar/gku1109 (2015).

13 Weis, F. *et al.* tmRNA-SmpB: a journey to the centre of the bacterial ribosome. *EMBO J* **29**, 3810-3818, doi:10.1038/emboj.2010.252 (2010).

14 Noeske, J. *et al.* High-resolution structure of the Escherichia coli ribosome. *Nat Struct Mol Biol* **22**, 336-341, doi:10.1038/nsmb.2994 (2015).
15 Pettersen, E. F. *et al.* UCSF Chimera--a visualization system for exploratory research and analysis. *J Comput Chem* **25**, 1605-1612, doi:10.1002/jcc.20084 (2004).
